# An ethnopharmacological evaluation of Navapind and Shahpur Virkanin district Sheikupura, Pakistan for their herbal medicines

**DOI:** 10.1186/s13002-017-0151-1

**Published:** 2017-05-08

**Authors:** Maria Zahoor, Zubaida Yousaf, Tahreem Aqsa, Manahil Haroon, Nadia Saleh, Arusa Aftab, Sadia Javed, Mouzma Qadeer, Habiba Ramazan

**Affiliations:** Department of Botany, Lahore College for Women University, Lahore, Pakistan

**Keywords:** Ethnobotany, Fabaceae, Medicinal plant, Poaceae, Traditional knowledge

## Abstract

**Background:**

The chief aim of this study was to enlist the ethnobotanical uses of wild plants in district Sheikhupura, province Punjab, Pakistan. Due to extreme geographical and climatic conditions, Pakistan has a great floral diversity. Plants have been used by the indigenous people for treatment of different ailments since long. They are still dependent on the plants for their domestic purposes. Moreover, plants are used as first aid to treat diverse ailments such as cold, cough, influenza, asthma, cancer, antidote, gastric and hepatic disorders. The traditional uses of medicinal plants lead to the discovery of natural drugs. This is first quantitative ethnobotanical documentation of medicinal plants in NavaPind and ShahpurVirkan district Sheikhupura, province Punjab, Pakistan.

**Methods:**

This ethnobotanical information was collected from about 400 informants including male and female. Sample size was determined by statistical formula. The informative data was based on semi-structured interviews, group discussions, Questionnaire and field visits. Then the data was analyzed by applying different quantitative indices such as Informant Consent Factor (ICF), Use value (UV), Relative Frequency of Citation (RFC), the Fidelity level (FL) and Jaccard Index (JI).

**Results:**

Almost 96 plants belonging to 34 families were reported. Most-frequently cited families were Poaceae (16 species) and Fabaceae (15 species). The most dominant life form was herbs (30.20%). The most-used plant parts were leaves (31.14%), followed by whole plant (24.59%), Most common mode of administration is extraction (81.25%). Generally herbal medicines were acquired from fresh plant material. Among all 54.16% plants were toxic, 31.25% were nontoxic, whereas the remaining 14.58% may be toxic or nontoxic because of their dual attitude. Almost 34 species were reported with their different medicinal uses as has been reported in literature.

**Conclusions:**

This ethnobotanical documentation revealed that the plants are still used by natives of rural areas in their day-to-day lives. This study provides basis for the conservation of local flora. Plants with high ICF, UV and FL can be further used for phytochemical and pharmacological studies. This documentation could provide baseline information which can be used to develop new plant-based commercial drugs.

**Electronic supplementary material:**

The online version of this article (doi:10.1186/s13002-017-0151-1) contains supplementary material, which is available to authorized users.

## Background

Ethnobotanical assessment of medicinal plant species is prerequisite for conservation, protection, and development of herbal drugs [1–3]. Plant and plant products play an important part in the material culture of many of the world’s native societies. Since the start of humanity extraction and processing of the medicinal plants to cure diseases is in practice. It also subsidizes economic uplifting of deprived areas [4]. Moreover, ethnobotanical studies indicated the importance of medicinal species within the local sociocultural context. This sort of study may support the socioeconomic conditions of an area; preserve the indigenous plant-based knowledge of the local communities and ultimately leads to conserve the global heritage [5, 6]. It provides a baseline for the discovery of new active compounds from the plants and being used directly as patent drugs. There are over 20,000 species of wild edible plants in the world, yet fewer than 20 species now provide 90% of our food [7]. The relationship between food and health has become significant increasingly. As nowadays consumers demand healthy, tasty and natural functional foods that have been grown in uncontaminated environment.

Wild resources of medicinal plants have been used by man for eras in conventional healing systems. Almost every country of the world follows herbs and some traditional medicine systems very efficiently. In developing countries about 65–80% of the population depends essentially on plants for their primary health care [8, 9]. In Indo-Pak Subcontinent, these herbs and traditional systems are known as Unani or Ayurveda system [10]. Pakistan is included in those countries where traditional Unani medicine is popularly practiced among the large segment of populations. The Unani medicine system originated in Greece was found by ancient Greek philosophers. It was documented and adopted by Muslims during the glorious period of Islamic civilization. Unani medicine system was brought to Indo–Pak subcontinent by Muslims scholars and practiced for centuries. It benefited from the Ayu system of medicine, which was an important component of Hindu civilization. Traditional Unani medicines were greatly depending on medicinal plants, apart from the animals and minerals [11, 12]. However, despite of the rich heritage of knowledge on the uses of plant drugs, little attention had been paid to document them in the country till the latter part of the nineteenths century. From 1996 to date, a number of ethnobotanical investigations in various geographical regions of Pakistan had been conducted [9, 13–16]. Pie and Manadhara [17] reported that in Himalayan ranges almost 70% of the medicinal plants and animals in the region consist of wild species. Globally, about 85% of all medications for primary health care are derived from plants [18]. So, there is need to explore either the areas have treasure of knowledge about the medicinal uses of plants and bring them into documentation to save ethnobotanical information and plant life [19].

Pakistan, comprises of nine major ecological zones and four phytogeographical zones, is bestowed with unique biodiversity. The country has about 6000 species of wild plants of which about 400–600 are considered to be medically important [20]. Till mid of the 20^th^ century, more than 80% of Pakistani population was dependent on ethnomedicines for their primary health care. Because of modernizing trends, now traditional system is largely experienced only in the rural areas. Natural resources and cultures are under the pressure of continuous change derived by these trends.

The village NavaPind and ShahpurVirkan district Sheikhupura are floristically quite rich tropical regions of Punjab. Ethnobotanical study of this area has never been conducted. The climate of the area is subjected to extreme variations. Wheat, Rice and Sugarcane are the main cash crops. Guavas, Strawberries and Citrus are grown at a larger scale in this district. Literacy rate of the villages is very low. Generally it is observed that most men in these areas are engaged in unskilled labor, while women are self-employed in petty trade of agriculture especially in the collection and trade of wild food and medicinal plants. Mostly plants are used for many purposes like food, shelter and therapeutic agents. However, lack of scientific knowledge about the useable parts, proper time of collection and wasteful methods of collection lead to mismanagement of these plants. So, the indigenous knowledge is going to be depleted, Hence ethnobotanical survey is planned for NavaPind and ShahpurVirkan district Sheikhupura, province Punjab to document the traditional uses of medicinal plants in the area before the information is lost. The main objective of present study was to document the indigenous therapeutic knowledge of plants. In addition to this it was also aimed to compile profile of medicinal plants by using quantitative indices like Use Values (UV), Relative Frequency Citation (RFC), Informant Consensus Factor (ICF), Fidelity Level (FL), Jacard Index (JI) and Relative Importance (RI) to evaluate available ethnobotanical data.

## Methods

### Study area

**Fig. 1:**
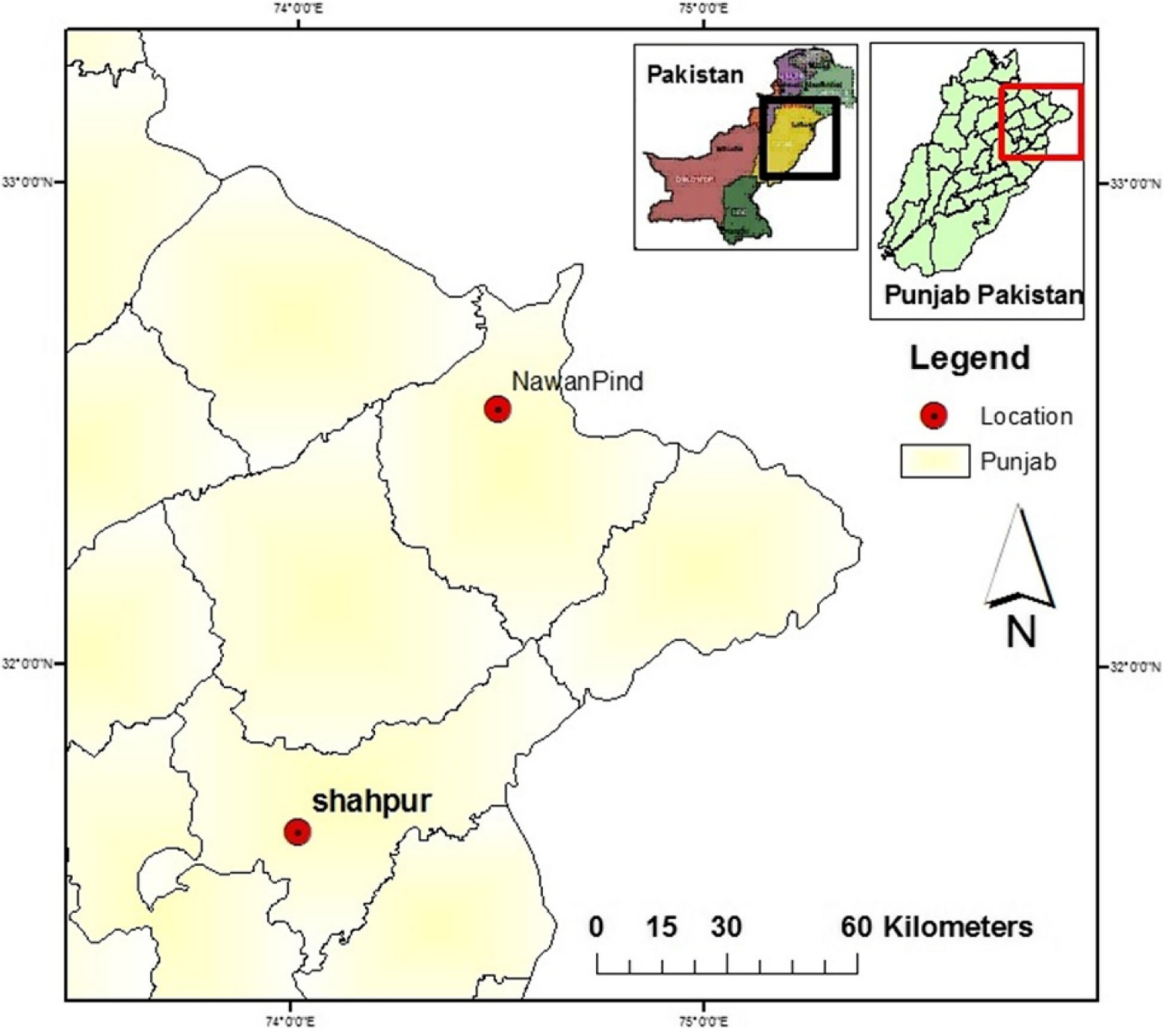
Map of NavaPind and Shahpur Virkan district Sheikhupura

Sheikhupura an industrial city is formerly known as Kot Dayal Das. It is part of province Punjab. District Sheikhupura bounded on the North by Gujranwala district, North-East by Narowal district, West by Nankana Sahib District and East by Lahore district [21] (Fig. 1). Its Southern boundary is formed by district Kasur. District Sheikhupura is spread over an area of 3,241 square kilometers and comprises four tehsils of: 1) Sheikhupura, 2) Ferozewala, 3) Sharaqpur, 4) Sharif Muridke [21]. According to the 1998 census of Pakistan, the district has a population of 3,321,029 of 25.25% is urban. Rest of population is resident of town and villages. The overall literacy rate of this region is 43.8% and it is ranked 15^th^ out of 34 district of Punjab in terms of literacy rate.

From mid of December to mid March the air is very damp, cold and light to moderate, rain falls at intervals. In April the temperature rises fast and two successive months are very hot [21]. Towards the end of June Monsoon conditions appear and during the following two and a half months spells of rainy weather. The average rainfall in the district is about 635 mm. The fauna and flora of the area include: Kikar, Pipal, Bohar, Eucalyptus, Popular and Sharin [21]. Major cash crops usually grown in this area are wheat, rice and sugarcane while minor crops are maize, millet, sorghum, cotton and mash.

From an ethnographic point of view, the region is occupied by two religious communities, Muslims in the majority and Christians in the minority. The main languages spoken in the District Sheikhupura include Urdu and Punjabi. There is a lack of satisfactory health-care facilities, thus resulting in high maternal mortality rate (MMR) and infant mortality rate (IMR). Almost all ethnic groups use herbal medicines to cure different diseases, and these groups are rich with indigenous knowledge regarding the use of medicinal plants.

### Experimentation

Experiment was conducted in two stages

- Field work
- Interview
- Questionnaire
- Inventory documentation
- Quantitative analyses of enthnobotanical data
- Field work:
  The Study was carried out by interviewing 400 informants including male, female and herbalists of the study area during November 2014 to June 2015. Sample size was determined by following Kadam and Bhalerao [22]. For plant material and traditional information collection, trips were arranged during winter, spring summer time to cover all seasonal variations in vegetation. Before visiting the area permission was taken from Chairman (representative of local government) of Shahpur Virkan and NavaPind. Moreover, Chairman also helped us to hire a local person as guide to facilitate the process of data collection. The methodology was adopted by following the work of Ahmad et al. [23], Qureshi and Raza Bhatti [15]; Qureshi et al. [13] Yaseen et.al. [3]. The survey was unique as emphasis was on both male and female members of different ages of the community. Local people were interviewed. Questionnaire was also used. But the majority of the people were not very well educated therefore interviewee filled the questionnaire herself after getting response form the informant.
- Inventory:
  The ethno botanical data obtained, checked and compared with existing literature. Hence the indigenous knowledge about the plant resources, religious and cultural aspect such as population density was also documented
*Ethnobotanical data Collection*
  The method used for data collection was based on semi-structured interviews, group discussions, and field visits. Interviews were conducted after obtaining informed consent (*I*C) from the interviewees. In many cases, a variety of techniques were used for selecting informants with traditional knowledge regarding the use of medicinal plants. The questionnaire (Additional file 1: Annex S1) used during the survey consisted of two parts: the first part was about the demographic data of the local informants, and the second part was about the medicinal plants. The ethnobotanical inventory consisted of local name of the plants, parts used, methods of preparation, mode of usage, and the diseases treated.
*Botanical Identifications*
  During field surveys, identification was mainly based on the local names of plants, with the help of local informants. For taxonomic confirmation, the Flora of Pakistan (http://www.efloras.org/index.aspx) was followed, whereas the International Plant Name Index (IPNI) (www.ipni.org) was also used to obtain the correct botanical name confirmation.
- *Quantitative Analyses of Ethnobotanical data*
  The documented ethnobotanical data was elucidated to define plant parts, and preparation methods. Various quantitative indices, including Use Value (UV), Relative Frequency of Citation (RFC), the Informant Consensus factor (ICF), and the Fidelity Level (FL), were applied.
  i. *Informant Consensus Factor (ICF)*
    ICF is calculated by the following formula, as cited in the literature [3, 24, 25]. ICF was applied to highlight the homogeneity of the information regarding particular types of ailment categories [18, 26].
    $$ \mathrm{I}\mathrm{C}\mathrm{F} = \left({\mathrm{N}}_{\mathrm{ur}}\hbox{--}\ {\mathrm{N}}_{\mathrm{t}}\right)/\left({\mathrm{N}}_{\mathrm{ur}}\hbox{--}\ 1\right) $$
    Where “N_ur_” mentions the number of use reports for a particular ailment category and “Nt” refers to the number of taxa used for a particular ailment category. The result of this consensus ranges from zero to 1. A high value (close to 1) specifies that taxa are used by the large proportion of the informants for a number of conditions related to that broad category, whereas the low value (close to 0) indicates that the plants are chosen randomly for a few or a single condition or that informants did not exchange information about the use of plants [18, 27–29].
  ii. *Fidelity Level (FL)*
    FL points out the preference shown for one species over others, for treating a particular ailment [18, 30]. The high FL confirms high usage of the plant species for a particular ailment, whereas low FL confirms a wide range of medicinal uses but with a low frequency for each ailment. It was calculated by the following formula:
    $$ \mathrm{F}\mathrm{L} = {\mathrm{I}}_{\mathrm{p}}/{\mathrm{I}}_{\mathrm{u}} \times 100; $$
    Where “I_p_” is the number of informants, provided information about use of species for a particular ailment category. Whereas “I_u_” is the number of informants stating the use of that plant for any ailment category. High FL value (near 100%) is obtained from plants for which almost all use reports mention using it in nearly the same way, whereas low FL value is obtained from plants that are used for many different purposes. Similarly, the high FL confirms high usage of the plant species for a particular ailment, whereas low FL confirms a wide range of medicinal uses but with a low frequency for each ailment.
  iii. *Use Value (UV)*
    The UV of reported species was calculated using the following formula [18, 24]:
    $$ \mathrm{U}\mathrm{V} = \mathrm{U}/\mathrm{n}; $$
    Where UV is the use value of species, “U” is the total number of use reports per species, and “n” represents the total number of informants interrogated for a given plant. UVs are higher if there are many use reports of a plant, implying that the plant is important, whereas they are near zero if there are few reports related to its use.
  iv. *Relative Frequency Citation (RFC)*
    The RFC was calculated using the following formula [18, 31].
    $$ \mathrm{R}\mathrm{F}\mathrm{C} = \mathrm{F}\mathrm{C}/\mathrm{N}\ \left(0 < \mathrm{RF}\mathrm{C} < 1\right): $$
    This relation displays the local prominence of every species, and it is calculated by dividing the FC, the number of informants reporting the use of the species divided by the total number of informers contributing in the survey (N), without bearing in mind the use-categories [2].
  v. *Relative Importance (RI)*
    RI was calculated using the following formula [18, 31]
    $$ \mathrm{R}\mathrm{I} = \left(\mathrm{PP} + \mathrm{AC}\right) \times 100/2; $$
    where PP stands for pharmacological properties, which indicate relative use reports, are calculated by dividing the number of use reports (UR) attributed to a species by the maximum number of use reports attributed to the most important species (the species with the highest number of use reports), and AC stands for ailments treated, which indicates the relative body systems treated. AC is calculated by dividing the number of body systems treated by a given species, by the maximum number of ailment categories treated by the species that are used most widely.
  vi. *Jacard Index (JI)*
    Ethnobotanists calculate the JI for comparison of documented data with previous published data collected from adjoining areas [18, 32]. JI was calculated using the following formula:
    $$ \mathrm{J}\mathrm{I} = \mathrm{c} \times 100/\left(\mathrm{a} + \mathrm{b}\right)\hbox{-} \mathrm{c} $$

where “a” is the number of species of the area A (the NavaPind or ShahpurVirkan); “b” is the number of species of the area B, which includes Thar desert, Pakistan; Alpine and Sub-alpine regions; District Mastung of Baluchistan; Makerwal and GullaKhel, Pakistan; Gilgit-Baltistan, Pakistan; Malakand, Pakistan; Lesser Himalayas-Pakistan; Cholistan Desert, Pakistan; Bhera, District Sargodha, Pakistan; Dir, Khyber Pakhtunkhwa, Pakistan; Pind Dadan Khan, district Jhelum, Punjab, Pakistan; Khushab, Punjab, Pakistan; Dir Kohistan valleys, KPK, Pakistan; Kabal, Swat District, KPK, Pakistan; Southern Rajasthan, India; Chitral valley, Pakistan; Mahal Kohistan (Khirthar National Park); Mianwali District Punjab, Pakistan; District Bagh, Azad Kashmir, Pakistan and “c” is the number of species common to both A and B.

## Results and discussion

### Demographic data

Eight field trips (to cover seasonal variations) were carried out to compile ethnobotanical data associated with uses of medicinal plants. The total duration of the field study was approximately 10 months, from November 2014 to June 2015. A total of 400 informants were interviewed. Among the 400 informants, mostly were indigenous people (Table 1). A large number of people were in the age of 30–40 years (36.5%) and less than 20 years (21%). Due to the lack of some educational facilities in that area, most of the informants were illiterate (50.25%) (Table 1). But some were educated showing that they had awareness about education. Many informants had completed their primary (24.75%) or secondary (10.25%) education. Some had completed even their secondary school certification (6.25%). Most of the informants spoke Punjabi and very often spoke Urdu. The majority of informants were female (71%) rather than male (29%). This may be because of female interviewer, hence females felt comfortable with her and talk freely.

**Table 1:** Demographic data of informants in NavaPind and Shahpur Virkan district Sheikhupura

Sr.no.	Variation	Category	No. of person	%age
1.	Informant category	Traditional health practitioner	0	0%
		Indigenous people	400	100%
2.	Gender	Male	116	29%
		Female	284	71%
3.	Age	Less than 20	85	21.25%
		20–30	41	10.25%
		30–40	146	36.5%
		40–50	77	19.25%
		50–60	40	10%
		60–70	7	1.75%
		70–80	2	0.5%
		More than 80	2	0.5%
4.	Educational background	Illiterate	201	50.25%
		Completed 5 years education	99	24.75%
		Completed 8 years education	41	10.25%
		Completed 10 years education	25	6.25%
		Completed 12 years education	17	4.25%
		Some under grade degree	10	2.5%
		Graduate	6	1.5%
		Master	1	0.25%

### Medicinal plant diversity

**Fig. 2:**
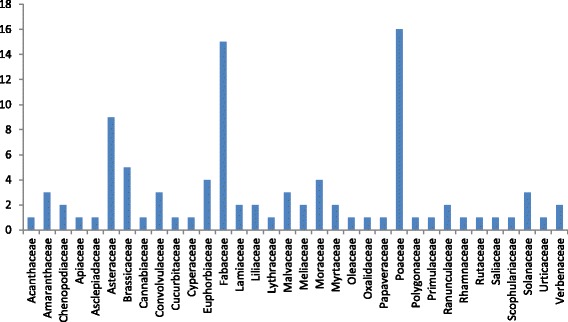
Plant families of medicinal plants

During ethnobotanical survey, 96 plant species were explored for medicinal properties (Fig. 2). The highest number of ethnobotanically used species were belonging to family Poaceae (16 species), followed by Fabaceae (15 species), Asteraceae (9 species), Brassicaceae (5 species), Euphorbiaceae and Moraceae (4 species), Amaranthaceae, Convolvulaceae, Malvaceae and Solanaceae (3 species/family). Other frequently used families were Chenopodiaceae, Lamiaceae, Liliaceae, Meliaceae, Myrtaceae, Ranunculaceae and Verbenaceae (2 species from each family). While Acanthaceae, Apiaceae, Asclepiadaceae, Cannabinaceae, Cucurbitaceae, Cyperaceae, Lythraceae, Oleaceae, Oxalidaceae, Papaveraceae, Polygonaceae, Primulaceae, Rhamnaceae, Rutaceae, Saliaceae, Scrophulariaceae, Urticaceae were represented by single species.

### Life forms

**Fig. 3:**
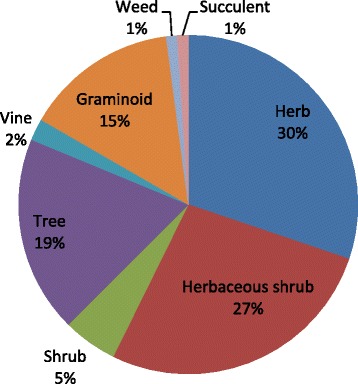
Life forms of medicinal plants

In this survey, herbs were documented with highest frequency (30.20%), then herbaceous shrubs (27.08%) which was followed by trees (18.75%), graminoids (14.58%), shrubs (5.20%), vines (2.08%), weeds and succulents (1.04% each) (Fig. 3). The possible reason for the dominance of herbs, might be the presence of bioactive compounds helping them to adapt the habitat. [1, 25, 33, 34]. This observation is supported by the previous study carried out in the other regions of Pakistan [18, 35]. While the shrubs (8.33%) and bushes (2.77%) have the lowest frequency. *Avena sativa* L., *Brassica campestris* L., *Triticum aestivum* L., *Coriandrum sativum* L. reported to cultivate in the study area for satisfication of the local need.

### Parts of medicinal plants used for curing diseases

**Fig. 4:**
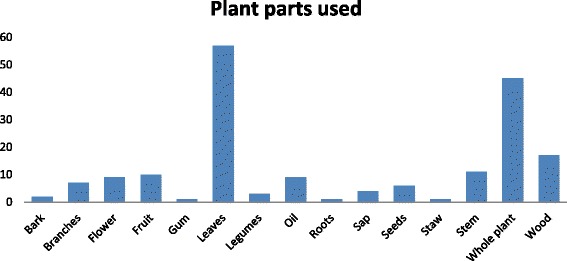
Plant part used (%)

In this survey, leaves (31.15%) were the dominant plant part used, followed by whole plant (24.59%), wood (9.29%), oil (4.92%) (Fig. 4). As leaves are easy to collect and require less effort than other parts such as root, seeds, flower and fruit [34], therefore they are frequently used for treatment of ailments. Another significant reason is that use of leaves can conserve the plant but the use of root can kill them [36, 37]. The lowest plant parts used were gum, oil, roots and straw (0.862% each). Moreover, mostly leaves were used for the preparation of herbal compositions [38–44]. This trend of plant part used is observed not only in NavaPind and Shahpur Virkan but throughout the province [45–48]. However, in other provinces particularly in KPK, whole plant utilization for curing various diseases is common practice [49].

In the present study difference in plant part used is also noticed. Although the medicinal use of species is already reported from other regions of Pakistan and South Asia but the important discovery is different plant part is used for treating the same diseases. *A. paniculata,* whole plant [47, 48] was reported before for the treatment of GIT problem and also used as blood purifier whereas in present it is discovered that flower and leaves can also be used to treat the same disease. Stem of *P. paniculata* was reported in previous study to minimize severe body pain [50] but this study reveals that whole plant can be used to relieve stomach pain. Similarly, shoot [45], seed [35] and fruit [51] of *C. sativum* were reported for to treat GIT problems, while present study describe that whole plant can be used for the same purpose. For *A. scoparia*, shoot and seed [52] were reported earlier for stomach complaints. But this study explored that whole plant can also be used for the same disease as well. Shoot extract, oil [45] and seeds [52] of *B. campestris* were reported as hair tonic but present study reveals that extract of leaves and flower can also be used for the same purpose. Shoot of *C. didymus* was explored in literature [45] as blood purifier whereas whole plant especially stem and leaves can also be used to treat the same disease.

Similarly shoot of *E. helioscopia,* was previously recommended for animal disease [45], whole plant as skin tonic [53], Stem and root as laxative and for cholera [54] were reported’ however present exploration indicated that leaves, sap and seeds can also be used for the same purpose. Effective of *A. arabica* leaves and fruit [55] was explained for dysentery whereas this study provides information that bark could be used for the same purpose. As far as *A. lebbeck,* bark, fruit and roots previous exploration concern, they were used to treat cough [48] but present study explained that leaves, seed and wood can be used also for the treatment of same cough. Different plant parts of *C. angustifolia* were in use for treating various diseases such as *l*eaves and roots for stomach disorder [3, 47] whereas branches, pods and seed can be used for stomach disorder as well. Stem of *M. indica* was reported in previous study as most plant part used for the treatment of stomach complaint [54]. However, according to present study findings whole plant can also be recommended for the same purpose. As leaves and flower of *V. thapsus*, were explored as for wound healing [11] while this study explained that seeds can also be used for wound healing.

### Toxicity of medicinal plants

**Fig. 5:**
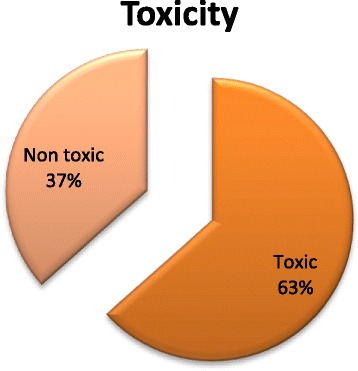
Toxicity of medicinal plants

In this study, the documented ethnomedicinal data was generally confined to medicinal usage, with some information on the toxicity of plants. The majority of plants (63%) were reported having toxic effect (Fig. 5). The majority of the informants stated that they use medicinal plants based on experience of their elders as they do not believe that the plants are toxic. According to some of the informants, plant-based recipes, when used in excessive dosage, may have severe toxic effects [47, 48]. The reporting of toxic effects of plants may provide useful information that should be taken into consideration by researchers in future investigation. It will also help them to determine the toxic compounds of plants for safe medicinal usage [56].

### Form and mode of utilization

**Fig. 6:**
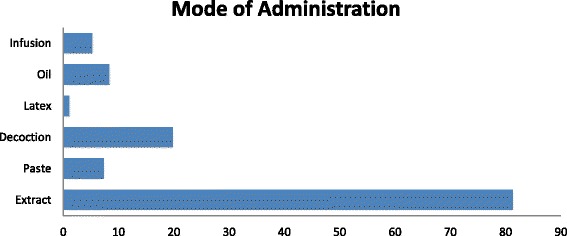
Mode of administration

In this survey, mostly plants were reported for internal applications. They were used either in the form of decoction (19.79%) or in the form of extract (81.25%) (Fig. 6). This form of utilization is common in other parts of Pakistan particularly and in World generally [35, 57, 58]. The decoction was made by boiling the plant parts in water [58–64]. While 44.44% were recommended for external use in the form of paste. The majorities of herbal medicines were prepared from fresh plant material rather than dried material.

### Quantitative analyses of ethnobotanical data

#### Informant Consensus Factor (ICF)

ICF was determined for 13 ailments (Respiratory diseases, GIT disease, Sexual disorder, Nail, skin, hair and teeth disorders, cardiac disorders, antidote, blood purifier, wound healing, fever cold and influenza disorders, Urinary disorders, Muscles disorders, glandular disorders and animals diseases) categories. The value of ICF for these 13 ailments was ranging from 0.02 to 0.82 (Table 2). The average ICF was 0.311. Commonly the value of ICF for disease treatment depends upon the availability of plant species in that area [65]. The highest value of ICF was reported for urinary diseases (0.82), sexual disease (0.68) and antidote (0.51). It showed that urinary diseases were the most common disease in the study area and most of the people had knowledge about its cure. They were using 7 different species (*Amaranthus viridis, Chenopodium album, Artemisia scoparia, Sonchus arvensis, Brassica nigra, Vicia fava, and Anagallis arvensis).* These seven species were not only used for urinary disease in this region but also reported from other regions of Pakistan like Semi-Tribal Area of Makerwal and Gulla Khel (Lying between Khyber Pakhtunkhwa and Punjab Provinces), Cholistan Desert and Thar Desert (Sindh). However these species used for curing cough and stomach disorders in Bhera, District Sargodha, Mastung district of Balochistan and Mountainous region of Gilgit-Baltistan [3, 49, 66] While the lowest value of ICF was for fever (0.02), muscles disorders (0.05) and glandular disorders (0.09) that may be due to lack of communication among the informants in the study area [65].

**Table 2:** ICF value of plants used for the treatment of various diseases

Category of diseases	No. of use reports	% Age of use reports	No. of taxa used	% Age of taxa	ICF
Respiratory diseases	2383	18.31	22	15.38	0.16
GIT diseases	3499	26.88	34	23.78	0.11
Sexual diseases	294	2.26	2	1.40	0.68
Nail, skin, hair, teeth disorders	945	7.26	18	12.59	0.45
Cardiac disorders	521	4.00	4	2.80	0.4
Antidote	378	2.90	7	4.90	0.51
Blood purifier	410	3.15	4	2.80	0.16
Wound healing	1397	10.73	13	9.09	0.16
Fever, cold, influenza disorders	1419	10.90	16	11.19	0.02
Urinary disorders	221	1.70	7	4.90	0.82
Muscles disorders	571	4.39	6	4.20	0.05
Glandular disorders	591	4.54	7	4.90	0.09
Animal disease	389	2.99	3	2.10	0.44

#### Uses Value (UV)

In this study, UV ranged from 0.005 to 0.07 (Table 3). The highest UV was recorded for *M. verticillata* (0.07), *A. farnesiana* and *C. occidentalis* (0.04 each). Plants with high UV were also used in many parts of Pakistan. To develop the herbal drug after pharmacological and phytochemical screening in the future, focus on the plant species with higher UV, enhance plant resources sustainability and conservation (Qureshi et al. [67]). While the lowest UV was reported for *C. sativum (*0.005), *T. tenellus* (0.0053) and *L. aphaca* (0.0055). Most of the informants were not familiar about these plant species and had little knowledge about their enthobotanical uses. Lower UV values indicated less knowledge about particular species in the study area. Same observations of plant uses were also made by Abbasi et al. [68]; Ahmad et al. [58]; Bano et al. [69] in their study areas. Although it was impossible to match quantitative data within the region particularly in district Sheikhupura because this is first quantitative ethnobotanical documented data in this region.

**Table 3:** Ethnobotanical uses of plants in NavaPind and Shahpur Virkan, Sheikhupura (Punjab), Pakistan

Sr. no.	Family	Local name	Life form	Habitat	Part used	Methods of preparation	Diseases treated	Toxicity	FL %	UV	RFC	R.I
1.	*Andrographis paniculata* (Burm.f.) Wall. ex Nees LCWU-15-01. Acanthaceae	Jungle charaita	Herb	Wild	Flowers/leaves	Extract	Cough, influenza, diarrhea, **blood purifier**	Non toxic	32.19	0.015	0.66	50.8
2.	*Amaranthus spinosus* L. LCWU-15-02 Amaranthaceae	kurand	Herb	Wild	Branches stem/leaves	Extract	Diarrhea, **antidote**, fever, fodder	No toxic	41.60	0.026	0.765	3.7
3.	*Amaranthus viridis* L. LCWU-15-03 Amaranthaceae	Chulai	Herb	Wild/cultivated	Leaves	Extract, decoction, paste	**Constipation**, skin tonic, antidote, diuretic, cooking	Non toxic	51.84	0.02	0.92	25.5
4.	*Chenopodium album* L. LCWU-15-04 Chenopodiaceae	Bathu	Herb	Wild/cultivated	Leaves/stem (whole plant)	Extract, decoction	**Laxative**, diuretic, cough, cooking	Non toxic	36.30	0.01	0.95	14.6
5.	*Chenopodium murale* L. LCWU-15-05 Chenopodiaceae	Jasag	Herb	Wild/cultivated	Whole plant	Extract, decoction	**Laxative**, cooking, Fodder, dye	Non toxic	27.77	0.02	0.94	5.9
6.	*Achyranthes aspera* (L.) Hill LCWU-15-06 Amaranthacea	Puth kanda	Herb	Wild/cultivated	Leaves	Decoction, extract, fresh	**Cough**, laxative, stomach complains, fodder, fuel	Toxic	12.35	0.02	0.68	7.5
7.	*Coriandrum sativum* L. LCWU-15-07 Apiaceae	Dhaniya	Herb	Wild/cultivated	Whole plant	Oil, extract	**Joint pain**, stomach complaints, cooking	Non toxic	14.55	0.005	0.91	29.7
8.	*Calotropis procera* R. Br. LCWU-15-08 Asclepiadaceae	Aak	Shrub	Wild	Leaves/sap milk	Latex, extract, decoction	**Asthma**, skin tonic, fever, fodder	Toxic	24.48	0.03	0.81	38
9.	*Artemisia scoparia* Waldst. & Kitt. LCWU-15-0902 Asteraceae	Char/Krund	Herb	Wild	Leaves/(Whole plant)	Oil, extract	**Diuretic**, stomach complains, fodder	Non toxic	8.09	0.013	0.77	7.3
10.	*Carthamus ticntorius* L. LCWU-15-10 Asteraceae	Jungle poli	Herbaceous	Wild	Whole plant	Extract	**Laxative**, wound healing, fodder	Toxic	3.09	0.0062	0.795	9.26
11.	*Conyza bonariensis* L. LCWU-15-1102 Asteraceae	Aflatoon/jungle boti	Herbaceous	Wild	Whole plant	Extract, oil	**Asthma**, diarrhea, ulcer, fodder	Toxic/Non toxic	37.33	0.014	0.635	8.83
12.	*Conyza erigeron* L. LCWU-15-1202 Asteraceae	Jungle kurand/jungle boti	Weed	Wild	Whole plant	Decoction	**Stomach disorder**, cold and cough, fever, fodder	Non toxic	9.905	0.013	0.81	8.83
13.	*Eclipta alba* (L.) Hassk. LCWU-15-13 Asteraceae	Jungle booti	Herb	Wild	Whole plant	Paste, extract	Liver tonic, hair tonic, **antidote of scorpio**, fodder	Non toxic	2.59	0.0075	0.665	5.87
14.	*Parthenium hysterophorus* L. LCWU-15-14 Asteraceae	Shah-tara/boti	Herbaceous	Wild	Whole plant/leaves	Extract	blood purifier, **back pain**, fodder, Decoration	Toxic	56.29	0.02	0.96	20.8
15.	*Silybum marianum* L. LCWU-15-1501 Asteraceae	Poli/boti	Herbaceous	Wild	Whole plant	Decoction, infusion	Anticancer, liver tonic, **wound healing**, fodder	Toxic	27.52	0.02	1.39	29.82
16.	*Sonchus arvensis* L. LCWU-15-1601 Asteraceae	Dodak	Herbaceous	Wild	Leaves/flower	Extract	Anti-kidney stone, **asthma**, cough, fodder	Toxic	13.74	0.012	0.87	13
17.	*Xanthium strumarium* L. LCWU-15-17 Asteraceae	Jungle booti	Herb	Wild	Leaves	Extract	Fever, **mother postnatal care**, fodder	Toxic	2.61	0.0074	0.67	4.28
18.	*Brassica compestris* L. LCWU-15-18 Brassicaceae	Sarson	Herb	Wild/cultivated	Leaves/flower	Extract, paste	**Skin tonic**, hair tonic, anti-cancerous	Non toxic	41.73	0.0075	0.7975	4.76
19.	*Brassica nigra* L. LCWU-15-19 Brassicaceae	Jungle sarson	Herbaceous	Wild	Whole plant	Extract, decoction	**Diuretic**, respiratory problems, fodder	Toxic if in **excess**	2.73	0.0081	0.61	20.45
20.	*Capsella bursapastoris* L. LCWU-15-20 Brassicaceae	Mirch boti	Herb	Wild	Whole plant	Decoction, infusion	Internal and external bleeding, **diarrhea**, fodder	Toxic	17.74	0.02	0.77	17.2
21.	*Coronopus didymus* (L.)Sm. LCWU-15-2003 Brassicaceae	Jungli pudina/jungle boti	Herb	Wild	Stem/leaves (whole plant)	Extract	**Asthma**, Blood purifier	Non toxic	13.96	0.015	0.7525	7.05
22.	*Sisymbrium irio* L. LCWU-15-96 Brassicaceae	Boti	Herb	Wild	Whole plant	Extract, powder	**Asthma**, fodder,	Non toxic	24.13	0.01	0.96	13
23.	*Cannabis sativa* L. LCWU-15-22 Cannabiaceae	Bhang	Herbaceous	Wild	Leaves	Extract, paste	**Stomach complaints**, Relaxant, Severe pains repellent	Toxic	42.31	0.021	0.945	3.7
24.	*Convolvulus arvensis* L. LCWU-15-23 Convolvulaceae	Lilly	Herbaceous	Wild	Leaves	Extract	Fodder, **animal fever**	Toxic	34.18	0.02	0.81	17.6
25.	*Cuscuta reflexa* Roxb. LCWU-15-24 Convolvulaceae	Amar-bail	Climber	Wild	Stem	Extract	**Wound healing**, Hair tonic	Toxic	20.42	0.0186	0.805	10.8
26.	*Poranopsis paniculata* (Roxb.)Roberty LCWU-15-25 Convolvulaceae	Gulu	Herb	Wild	Whole plant	Extract,	Decoration, **stomach pain**, diarrhea, wound healing	Toxic to animals	27.65	0.007	0.69	29.57
27.	*Cucurbita pepo* L. LCWU-15-2601 Cucurbitaceae	Chibbar booti	Vine	Wild/cultivated	Leaves/flower/fruit/whole plant	Extract	**Skin tonic**, cardiac problem, stomach disorder, Cooking,	Toxic/Non toxic	20.75	0.025	0.60	33.83
28.	*Schoenoplectus supinus* (L.)Pall. LCWU-15-27 Cyperaceae	Dala	Herb	Wild	Flower/leaves	–	**Fodder, animal cough**	Non toxic	2.70	0.0071	0.695	
29.	*Euphorbia helioscopia* L. LCWU-15-28 Euphorbiaceae	Dhdtar boti	Herbaceous	Wild	Leaves/sap/seeds	Extract, decoction	Anticancerous, **cholera**	Toxic in excess	54.68	0.0136	0.695	50.8
30.	*Euphorbia hirta* L. LCWU-15-29 Euphorbiaceae	Boti	Herb	Wild	Whole plant	**–**	**Fodder**	Toxic	2.64	0.01	0.98	
31.	*Euphorbia thymifolia* L. LCWU-15-30 Euphorbiaceae	It-sit	Herb	Wild	Whole plant	**–**	**Fodder**	Toxic	2.55	0.0152	0.655	
32.	*Ricinus communis* L. LCWU-15-3101 Euphorbiaceae	Rind/Rnola	Herbaceous	Wild/cultivated	Leaves/seeds/whole plant	Extract	**Wound healing**, fuel, hair tonic	Seed are toxic	15.53	0.012	0.65	8.83
33.	*Sapium sebiferum* L. LCWU-15-32 Fabaceae	Jungli Tahli	Tree	Wild	Leaves/wood	Decoction	**Antidote**, diuretic, Fodder, fuel, furniture	Toxic/non toxic	9.94	0.01	0.85	
34.	*Acacia nilotica* (Linn.) Delile. LCWU-15-93 Fabaceae	Jawa	Herbaceous	Wild	Leaves	**–**	**Fodder**	Non toxic	1.81	0.007	0.68	
35.	*Acacia arabica* Willd. LCWU-15-3301 Fabaceae	Desi kikkar	Tree	Wild	Bark	Decoction	Diarrhea, **Skin tonic**, roofing, fuel, fodder	Non toxic	19.35	0.015	0.635	20.8
36.	*Acacia farnesiana* L. LCWU-15-34 Fabaceae	Kabli kikar	Tree	Wild	Wood/flower/leaves	Extract	**Diarrhea**, scent, fuel, furniture,	Non toxic	16.48	0.04	0.65	9
37.	*Albizia lebbeck* (L.) Benth. LCWU-15-3501 Fabaceae	Shireen	Tree	Wild	Leaves/seed/wood	Extract	Fuel, **wound healing**, Cough, burning, postnatal care	Non toxic	33.22	0.02	0.66	11.15
38.	*Cassia angustifolia* L. LCWU-15-3602 Fabaceae	Sukh chain	Tree	Wild	Branches/pods/seeds	Extract	**Purgative**, hair tonic, teeth tonic	Toxic/Non toxic	64.33	0.02	0.87	13
39.	*Cassia fistula* L. LCWU-15-37 Fabaceae	Amaltas	Tree	Wild	Leaves/ legumes	Infusion, extract	**Laxative**, fodder, cough, stomach pain	Toxic in excess	21.39	0.02	0.77	7.83
40.	*Cassia occidentalis* L. LCWU-15-38 Fabaceae	Boti	Herb	Wild	Wood/leaves	Extract	Stomach disorder, **laxative**, fuel, roofing, bitter taste	Toxic	19.45	0.04	0.72	8.83
41.	*Dalbergia sissoo* Roxb. ex DC. LCWU-15-3901 Fabaceae	Tali	Tree	Wild	Wood/leaves	Extract	**Wound healing**, hair tonic, fodder, fuel, furniture	Toxic/non toxic	34.64	0.025	0.91	8.83
42.	*Indigofera linifolia* (L.f) Retz. LCWU-15-40 Fabaceae	Gorakh pan/jungle boti	Herbaceous	Wild	Whole plant	Extract	Stomach complaints, blood purifier, **liver problems**, fodder, obtain dye	Toxic to some extent	55.13	0.02	0.722	75.8
43.	*Lathyrus aphaca* L*.* LCWU-15-41 Fabaceae	Jungli matter/gangla	Herbaceous	Wild	Leaves	–	**Fodder**	Toxic/non toxic	3.52	0.0055	0.905	
44.	*Melilotus indica* L. LCWU-15-4202 Fabaceae	Jungle meeni or meena/Jungli shatala	Herbaceous	Wild	Leaves/whole plant	Extract	Laxative, **diarrhea**, cooking, fodder, bitter taste, skin tonic	Toxic to some extent	18.17	0.02	0.78	8.58
45.	*Mimosa pudica* L. LCWU-15-94 Fabaceae	Chijan	Herb	Wild	Leaves	Extract	**Antidote**, wound healing, diarrhea, fuel, fodder	Toxic in excess	4.44	0.01	0.68	
46.	*Tamarindus indica* L. LCWU-15-4301 Fabaceae	Imli/khatti imli	Tree	Wild	Branchesstem/leaves/fruit/whole plant	Extract	Malaria, fever, bitter taste, **Laxative**, burning	Non toxic	39.61	0.03	0.83	27.1
47.	*Vicia faba* L. LCWU-15-4402 Fabaceae	Jungli cheraal/Phalya/Arari	Herbaceous	Wild	Legumes/leaves	Extract	**Diuretic**, fodder, food, soap	Non toxic	43.04	0.02	0.86	25.8
48.	*Ocimum basilicum* L. LCWU-15-45 Lamiaceae	Niazbo	Herb	Wild	Leaves	Extract	Scent, flavoring, **cough**, headache, diarrhea	Non toxic	37.96	0.0176	0.85	
49.	*Ocimum sanctum* L. LCWU-15-4601 Lamiaceae	Jungle booti	Herbaceous	Wild	Leaves/whole plant	Powder, extract, oil	Appetizer, mosquito repellent, fodder, **fever**, cough	Toxic/non toxic	11.50	0.008	0.798	14.7
50.	*Allium roylei* Strearn. LCWU-15-47 Liliaceae	Bola ganda	Herbaceous	Wild	Leaves	Oil, extract	**Cholera**, curative, flavoring	Non toxic	62.03	0.0163	0.915	25.8
51.	*Aloe vera* (L.) Burm. f. LCWU-15-48 Asphodelaceae	Kwal qandal	Succulent plant	Wild	Leaves	Oil, extract	Skin tonic, cough, hair tonic, **fever**	Non toxic	18.80	0.03	0.70	30.5
52.	*Lawsonia Inermis* L. LCWU-15-49 Lythraceae	Mehndi	Shrub	Wild	Leaves/roots	Paste, extract	**Wound healing**, hair tonic	Non toxic	28.04	0.0213	0.935	10.8
53.	*Malva verticillata* L*.* LCWU-15-50 Malvaceae	Booti	Herb	Wild	Whole plant	Paste	**Fodder**, animal ulcer	Non toxic	2.51	0.07	0.645	
54.	*Malva indica* LCWU-15-51 Malvaceae	Sonchal booti/jungle boti	Herbaceous	Wild	Whole plant	Decoction, extract	**Cough**, cooking, fodder	Toxic in excess	11.26	0.011	0.628	7.04
55.	*Malvastrum coromandelianum* L. LCWU-15-52 Malvaceae	Boti	Herbaceous	Wild	Whole plant	Extract	Fodder, **wound healing**	Toxic	16.06	0.02	0.60	8.83
56.	*Azadirachta indica* A. Juss LCWU-15-53 Meliaceae	Neem	Tree	Wild	Leaves/wood	Extract, oil	Fodder, **skin tonic**, fuel	Toxic to some extend	20.62	0.03	0.79	10.5
57.	*Melia azadirachta* L. LCWU-15-5402 Meliaceae	Bakain/dhareek	Tree	Wild	Leaves/wood/seed	Extract	**Wound healing**, cough, fuel, furniture, fodder	Seeds are toxic	22.04	0.03	0.94	9.22
58.	*Ficus sarmentosa* Bush: Ham. Ex J.E. Smith LCWU-15-95 Moraceae	Walaiti bohr	Tree	Wild	Wood/leaves	Decoction	**Fuel**, fodder	Toxic	6.89	0.01	0.85	
59.	*Ficus benghalensis* L. LCWU-15-5501 Moraceae	Borh/desi borh	Tree	Wild	Leaves/sap milk/fruit/wood	Decoction, extract	**Influenza,** diarrhea, fuel, decoction	Toxic to some extend	40.06	0.03	0.85	29.82
60.	*Ficus religiosa* L. LCWU-15-56 Moraceae	Peepal	Tree	Wild	Wood/leaves/bark	Decoction, extract	Asthma, diarrhea fuel, fodder, ulcer, molar pain, **cardiac problem**	Toxic in excess	28.33	0.03	0.902	19.4
61.	*Morus alba* L. LCWU-15-5701 Moraceae	Shehtoot/Toot	Tree	Wild/cultivated	Wood/fruit/branches/leaves	Extract	**Cough**, fodder, respiratory disorders	Sap is toxic	13.58	0.02	0.98	7.64
62.	*Eucalyptus globulus* Labill. LCWU-15-58 Myrtaceae	Sufaida	Tree	Wild	Leaves/wood/oil	Extract, infusion	**Influenza**, skin tonic, Fuel, furniture, stomach disorder	Toxic in excess	31.32	0.02	0.83	17.32
63.	*Syzygium cumini* L. LCWU-15-59 Myrtaceae	Jaman	Tree	Wild	Fruit/wood/leaves	Extract	**Heart problem**, diarrhea Fruit, fuel, fodder,	Non toxic	24.76	0.03	0.98	25.5
64.	*Jasminum nudiflorum* Lindl. LCWU-15-6002 Oleaceae	Chasko	Shrub	Wild	Leaves/flower/fruit	Extract	Vegetable, **stomach pain**, Cooking,	Toxic	9.71	0.03	0.59	
65.	*Oxalis corniculata* L. LCWU-15-6101 Oxalidaceae	Khatmal booti/jungle boti	Herb	Wild	Whole plant	Extract	**Antidote**, stomach disorder, cooking, fodder	Toxic	38.35	0.012	0.773	8.83
66.	*Fumaria indica* Linn. LCWU-15-6202 Papaveraceae	Soye/Papra	Herb	Wild	Branches/stem/leaves/whole plant	Extract	**Laxative**, fodder	Non toxic	11.04	0.01	0.83	3.7
67.	*Acrachne racemosa* (Roem&schult) LCWU-15-63 Poaceae	Jungle ghaas	Grass	Wild	Whole plant	Decoction	**Fodder**	Toxic on skin	3.17	0.0061	0.815	
68.	*Asthenatherum forkalii* (Vahl)Nexski. LCWU-15-64 Poaceae	Jungle ghass	Grass	Wild	Whole plant	Extract	**Fodder**, **animal fever**	Toxic/non toxic	3.27	0.0059	0.84	
69.	*Avena sativa* L. LCWU-15-65 Poaceae	Jo	Herb	Wild	Staw	Extract	**Postnatal care**	Toxic to some extend	51.40	0.01	0.945	5.3
70.	*Cenchrus setigerus* Vahl. LCWU-15-66 Poaceae	Jungli boti	Grass	Wild	Whole plant	Extract	**Fodder**	Non toxic	2.11	0.006	0.79	
71.	*Chloris barbata* Sw. LCWU-15-67 Poaceae	Jungle boti	Herb	Wild	Whole plant		Fodder, **skin disorder**	Toxic	22.70	0.01	0.78	10.5
72.	*Chloris virgata* Sw. LCWU-15-68 Poaceae	Boti	Grass	Wild	Whole plant		Fodder, wound healing, **malaria**	Toxic	50.09	0.02	0.71	25.5
73.	*Chrysopogon aucheri* Bioss LCWU-15-69 Poaceae	Goad ghass	Grass	Wild	Whole plant		Fodder, **fuel**	Toxic	5.26	0.01	0.73	
74.	*Cynodon dactylon* (L.) Pers. LCWU-15-70 Poaceae	Ghaas	Grass	Wild	Whole plant	Extract	Stomach complaint, **constipation**, antidote for snake and dogs, fodder	Non toxic	50.81	0.02	0.83	3.7
75.	*Digitaria ciliaris* (Retz.)Koel. LCWU-15-71 Poaceae	Jungle ghass	Grass	Wild	Whole plant		**Fodder, animal stomach disorder**	Toxic	3.29	0.0059	0.84	
76.	*Digitaria nodosa* Parl. LCWU-15-72 Poaceae	Swank	Grass	Wild	Whole plant	Fresh, dry	**Fodder, blood purification**	Toxic	3.43	0.011	0.88	
77.	*Eleusine indica* (L.)Gaertn. LCWU-15-73 Poaceae	Jungle ghaas	Grass	Wild	Whole plant	Extract	**Fodder**	Toxic/non toxic	3.04	0.0064	0.78	
78.	*Pennisetum divisum* (Fosslk.ex.J.F.Gmel) LCWU-15-74 Poaceae	Jungle ghaas	Grass	Wild	Whole plant	Extract	**Fodder**	Non toxic	3.39	0.0057	0.87	
79.	*Phragmites karka* (Retz.) Trin. Ex Steud. LCWU-15-75 Poaceae	Nur	Grass	Wild	Wood/stem	Extract	**Cardiac problem**, fuel	Toxic/non toxic	23.80	0.01	0.62	13
80.	*Saccharum bengalensis* L. LCWU-15-76 Poaceae	Kana	Grass	Wild	Wood	Paste, extract	**Animal disease** roofing, fuel, pre/post natal care	Toxic	35.46	0.02	0.84	17.16
81.	*Tetrapogon tenellus* (Roxb.)Chiov. LCWU-15-77 Poaceae	Dumbi seeti	Grass	Wild	Whole plant	Extract	Fodder, **cough**	Toxic	3.64	0.0053	0.935	
82.	*Triticum aestivum* L. LCWU-15-78 Poaceae	Joi	Grass	Wild	Whole plant	Extract	Fodder, anticancerous, **gastrointestinal disease**	Toxic/non toxic	3.19	0.01	0.985	8.9
83.	*Rumex chalepensis* Mill. LCWU-15-79 Polygonaceae	Boti	Herbaceous	Wild	Whole plant	Extract	**Fodder**	Toxic	2.05	0.01	0.76	
84.	*Anagallis arvensis* L. LCWU-15-8001 Primulaceae	Jungli golo/Matri	Herbaceous	Wild	Leaves/wood (whole plant)	Extract	Fodder, **skin tonic, Diuretic**, hair tonic	Toxic/non toxic	31.44	0.02	0.855	18.15
85.	*Ranunculus muricatus* L. LCWU-15-8101 Ranunculaceae	Jungli dhaniya/boti	Herbaceous	Wild	Leaves/whole plant	Crude extract	Fodder, fever**, asthma, Fodder**	Toxic	14.5	0.01	0.738	25.5
86.	*Ranunculus repens* L. LCWU-15-82 Ranunculaceae	Jungle jugnoo	Herbaceous	Wild	Leaves/flower	Extract	**Fodder**	Toxic	2.47	0.007	0.635	
87.	*Zizyphus jujuba* (L.)Lam. LCWU-15-8301 Rhamnaceae	Ber	Tree	Wild	Leaves/gum/fruit/wood/leaves	Extract, oil	Fruit, furniture, digestion, skin tonic, joints pain, hair tonic, **backache**, burning	Toxic/non toxic	51.75	0.03	0.97	30.65
88.	*Murraya koenigii* L. LCWU-15-84 Rutaceae	Marva	Tree	Wild	Branches/stem/leaves	Extract	**Stomach diseases**, wound healing, vomiting, scent, decoration	Toxic/non toxic	34.86	0.017	0.87	14.5
89.	*Salix lanata* L. LCWU-15-85 Salicaceae	Boti	Shrub	Wild	Whole plant	Extract	Fever, **reduce internal pain**, fodder, bitter taste	Toxic	14.93	0.02	0.57	26.98
90.	*Verbascum thapsus* L. LCWU-15-86 Scrophulariaceae	Geedar tabbaco	Herb	Wild	Seeds	Extract, infusion	**Male disease**, internal bleeding	Toxic to some extent	42.17	0.008	0.62	25.8
91.	*Lycium barbarum* L. LCWU-15-87 Solanaceae	Kashni	Herbaceous	Wild	Leaves	Extract	A**nticancerous**, asthma, scent, fodder	Non toxic	55.71	0.022	0.895	33.8
92.	*Solanum americanum* Mill. LCWU-15-88 Solanaceae	Peelak	Herb	Wild	Leaves/stem	Extract	**Joints pain**, cooking, scent, vegetable, fodder	Toxic in excess	66.14	0.03	0.85	10.8
93.	*Withania somnifera* L. LCWU-15-89 Solanaceae	Aak-Singh	Herbaceous	Wild	Leaves/flower/fruit	Extract	Lung disorder, animal disease, asthma, **male disease**	Toxic in excess	44.09	0.0085	0.79	42.32
94.	*Urtica dioca* L. LCWU-15-90 Urticaceae	Kharish booti	Herbaceous	Wild	Stem/leaves	Extract	**Joints pain**, cold and flu, liver disease	Toxic in excess	16.33	0.010	0.945	10.8
95.	*Lantana camara* L. LCWU-15-9101 Verbenaceae	Cingii	Shrub	Wild	Leaves	Extract	Fodder, snake bite, **cold and flu**	Toxic	48.16	0.02	0.70	50.5
96.	*Phyla nodiflora* L. LCWU-15-92 Verbenaceae	Bakan	Herb	Wild	Branches/stem/leaves	Extract, decoction	**Fodder**	Toxic	2.08	0.018	0.535	

*FL* Fidelity Level, *RFC* Relative Frequency of Citation, *UV* Use Value, *RI* Relative Importance

Bold Uses/Ailment treated = Main use of plant on which FL (%) is based

#### Relative Frequency of Citation (RFC)

The value of RFC ranged from 0.535 to 1.39. (Table 3). The highest value of RFC present in *S. marianum* (1.39), *T. aestivum* (0.985), *E. hirta*, *M. alba* and *S. cumini* (0.98 each). It can be seen that plants with highest RFC are the most common plant in that region and majority people agreed by its medicinal value [35]. While the lowest value of RFC was present in *P. nodiflora* (0.535), *S. lanata* (0.57) and *J. nudiflorum* (0.59). RFC was compared with previous studies including Yaseen et al. [18]; Xavier et al. [70]; Bibi et al. [35].

#### Fidelity Level (FL)

In present study the FL ranged from 1.81 to 66.14% (Table 3). The higher the FL value the more would be use of plant [18]. The highest value of FL was present in *S. americanum* (66.14%), *C. angustifolia* (64.33%) and *A. roylei* (62.03%). These plants were used by informants for disease cure and other purposes. The choice of informants dealing with the specific ailments indicated maximum value of FL (Rajakumar and Shivanna, [65]; Islam, [71]. While lowest FL value was in *A. nilotica.* (1.81%), *R. chalepensis* (2.05%) and *P. nodiflora* (2.08%).

#### Comparison with other studies in neighboring regions

To compare our documented data regarding important plant species in the NavaPind and ShahpurVirkan ethnobotanical data from 17 published studies of neighboring regions (Table 4) were cited. The ethnobotanical studies of India (because Pakistan and India both are Asian countries and sharing same physical and biological conditions with minute differences) were also included for the comparative studies. The percentage similarity for uses of the total comparative study varied from 14.28% [72] to 66.66% [73]. While the percentage of dissimilarity for uses of the total comparative study ranged from 85.71% [72] to 6.66% [18]. The highest value of JI was calculated to be 32.98 [45], followed by 19.58 [49]. Whereas the lowest value of JI was 2.29 [66], followed by 4.43 [72]. From this comparison, it can be calculated that more similarity means the sharing of same flora and cross-cultural exchange of medicinal plant knowledge in the past.

**Table 4:** Comparison of medicinal plant species of NavaPind/Shahpur Virkan and Allied areas

	Area	Study year	No. of recorded plant species	Total species common in both area	Species enlisted only in study area	Species enlisted only in aligned area	Plant with similar uses (%)	Plant with dissimilar uses (%)	JI	Citation
1.	Thar desert, Pakistan	2015	87	16	81	72	60	6.66	10.8	[18]
3.	District Mastung of Balochistan	2014	102	12	84	90	16.66	83.33	7.40	[35]
4.	Makerwal & Gulla Khel, Pakistan	2013	131	28	68	103	35.71	64.2	19.58	[49]
5.	Gilgit-Baltistan, Pakistan	2013	49	7	89	42	57.14	42.85	5.64	[93]
6.	Malakand, Pakistan	2013	92	14	82	78	42.85	57.14	9.58	[11]
7.	Lesser Himalayas-Pakistan	2013	45	8	88	37	37.5	62.5	6.83	[68]
8.	Cholistan Desert, Pakistan	2012	90	4	92	86	50	50	2.29	[66]
9.	Bhera, District Sargodha, Pakistan	2012	97	32	64	65	37.5	62.5	32.98	[45]
10.	Dir, Khyber Pakhtunkhwa, Pakistan	2012	67	18	78	49	33.33	66.66	16.51	[53]
11.	Pind Dadan Khan, district Jhelum, Punjab, Pakistan	2011	69	14	82	55	35.71	64.28	11.38	[46]
12.	Khushab, Punjab, Pakistan	2011	48	15	81	33	26.66	73.33	15.15	[54]
13.	Dir Kohistan valleys, KPK, Pakistan	2011	65	9	87	56	66.66	33.33	6.71	[73]
14.	Kabal, Swat District, KPK, Pakistan	2011	140	24	72	116	25	75	14.63	[9]
15.	Southern Rajasthan, India	2010	31	5	91	26	40	60	4.46	[98]
16.	Chitral valley, Pakistan	2009	83	7	89	76	14.28	85.71	4.43	[99]
17.	Mahal Kohistan (Khirthar National Park)	2007	50	11	86	40	36.36	63.63	8.62	[76]
	Average		73.29	13.17	77.29	60.23	36.19	55.94	10.41	

Man learnt to cure health problems with plant and plant products by trails and errors. With the passage of time this knowledge got the shape of traditional medicinal system. This system got transferred from one generation to other generation orally without any documentation. Hence in last few decade lack of interest by generation created gap in knowledge transformation and highlighted the need to bring information about traditional medicine into documentation.

In 1950 Hocking reported that almost 84% of Pakistan population relied on natural medicinal resources for their health care [74]. With reference to Pakistan first enthnobotanical study was made by Chaudhary from West Pakistan [75]. According to the survey, almost 1500 medicinal plant species were being used to cure many aliments. Even now modern pharmacopoeia contains at least 25% drug which can be derived from natural resources. The scope of medicinal plants can revolutionize the lives of the people in the rural areas of Pakistan and also increase the socio-cultural values of that area of Pakistan. With the opening of new era, ethnobotany provides a solid vision of plant resources through which new targeting active compounds can be developed and can be used for every major and minor disease. diversity richness will provide germplam, leading to more targeting compounds.

The information presented in this document was collected from reliable informants based on personal observation. Different plant recipes were used to cure ailments. This is notable that most of the remedies consist of a single plant species. However, in other cases, one plant species is used in combination of other plant species to cure ailments like *Cassia fistula and T, indica.* One teaspoon Amaltas fruit pulp and 1 teaspoon of tamarind in one cup of water left overnight mashed and strained is used for stomach problem, Similarly, 1 plant species can be used more than one disease like *Amaranthus spinosus* is used as antidote and as well as for constipation. Moreover, a single ailment can be treated with a list of plant species like asthma can be cured (Additional file 1: Table S2).

Results of present study revealed that 12 out of 96 species were first time explored for the medicinal values. These species are *C. tinctorius*, *C. erigeron*, *S. supinus*, *O. sanctum*, *A. roylei, M. verticillata, M. indica, J. nudiflorum, A. forkalii, D. ciliaris, D. nodosa and T. tenellus*. These species belong to seven different families. These species have not been reported for their medicinal properties from any region of the Pakistan and South Asia before this report [9, 13–16, 28, 76]. Four species (*A. forkalii, D. ciliaris, D. nodosa and T. tenellus*) belong to family Poaceae [77]. This family is well known for food and fodder species. Members of this family are rich in phytochemicals like phenol, alkaloid, coumarins, glycosides, lignin, quinones, saponins, steroids, tannins, terpenoids and flavonoids [78]. Therefore they have been in used for treatment of various human and animals ailments. These four species are not only used as fodder but also for the treatment of animal fever, animal stomach disorders, blood purification and animal cough respectively. This information may lead toward the discovery of new fodder sources. They may also use for the exploration of number of phytochemicals to give another alternative way to compensate the drug needs. That’s why; this finding will help many pharmacologists in near future to determine many new pharmacologically active constituents from these plant species.

Two species (*M. verticillata and M. indica*) belong to family Malvaceae. This family is characterized by large number of phytochemicals including tannins, polysaccharides, coumarins, flavonoids, malvin, folic acid, and terpenoids, Vitamin A, Vitamin C and Vitamin E [79]. These phytochemicals make them suitable for the treatment of various human and animal ailments. These two species are not only used as fodder but can also be used for the treatment of various ailments including animal ulcer and cough respectively. Similarly, two species (*C. tinctorius and C. erigeron*) belong to family Asteraceae. This family is rich in many active compounds like saponin, glycosides, steroids, tannins, diterpenoids, tritepenoids and Flavonoids [80]. That’s why; the members of this family are being used for the ailments of human and animals as well. These two species are used for the treatment of GIT and respiratory disorders respectively in our study area. One species (*O. sanctum*) belongs to family Lamiaceae. Most of the members of this family are well known of having many phytochemicals (alkaloids, tannins, flavonoids, terpenoids and carbohydrates) [81]. They are used for the ailments of human and animals as well. This species is reported for the treatment of fever and cough. This could be an addition into herbs used for the treatment of fever and cough.

*A. roylei* is among the 12 species reported first time from present study area for their medicinal uses. This species belongs to family Liliaceae. Phytochemical analysis of other member of this family indicates the presences of phenols, flavonoids, tannins, saponins, steroids, terpenoids [82]. So, they are medicinally used for the treatment of cholera. *A. roylei* is reported for curing cholera. *J. nudiflorum* belongs to family Oleaceae. This family is characterized by alkaloids, glycosides, carbohydrates, anthraquinone, steroids, saponins, flavonoids, tannins and phenols [81]. It can be predicted that *J. nudiflorum* might be rich in these phytochemicals. This species is an effective herbal remedy to relieve stomach pain. *S. supinus* is belongs to family Cyperaceae. Based on previous literature [17] it could be predicted that *S. supinus* is rich in alkaloids, saponins, phenolic, flavonoids (Bhardwaj et al. [83]). This species is used for treating animal cough in NavaPind and Shahpur Virkan. This medical use of species is not report earlier [28, 76, 84]. This species could be further explored for the discovery of new drugs and active phytochemicals.

Present investigation provides baseline information to screen out biological activities of these valuable plants in order to develop new antiseptic and insecticidal medicines from plant origin. This can be possible when focus on the different medicinal uses of a plant species which are not reported in literature before. There were 96 species reported from NavaPind and Shahpur Virkan, Shiekhupura, Pakistan. To compare their medicinal uses with previous research work in Pakistan and neighboring country, almost 34 plant species were found that has been mentioned as herbal medicines but in the present study new medicinal uses were reported. In the study area *A. paniculata* is being used for cough and influenza while in literature, it was reported for malarial fever and liver complaints [47, 48]. From Shahpur Virkan and NavaPind *C. sativum* is reported for joints pain while in previous study it was reported as diuretic and for other digestive problems [35, 51]. Present study indicated that *A. scoparia* is diuretic in nature however it has been reported for earache, cardiac problem, fever and blood pressure [9, 54, 85]. Indigenous people of NavaPind and Shahpur Virkan are being using *C. bonariensis* for asthma and ulcer but in previous study it was reported for hemorrhage and diuretic [9, 86]. *E. alba* is discovered to be used as antidote of scorpio while previous researches indicated its use as laxative [48]. *P. hysterophorus* is used as blood purifier and backache, whereas in literature it was used in dysentery, fever and toothache [13, 14, 85, 86]. *S. marianum* is used as anticancer and wound healing but in literature it was used for Tuberculosis [58]. Another important discovery made during this study was use of *B. campestris* as anticancer while in previous study it was used for body massage and ulcer problems [52]. *C. didymus* is used for asthma while comparing to previous study it is used in rheumatism and bone disorder [48]. *P. paniculata* is used for stomach pain and wound healing while it was reported to minimize severe pain in the body [87]. *C. pepo* is used as skin tonic but according to the previous reports it was used as liver tonic [11]. *E. helioscopia* member of family Euphorbiacae expected to have large number of alkaloid is used for cancer and cholera treatment but in the study of Jandool Valley and Pind Dadan Khan, it was reported as laxative, skin tonic and respiratory disorders [88, 89].

*A. arabica* is an endangered species of Pakistan, used as skin tonic. Previous literature showed its efficacy for dysentery and cough [55]. *A. farnesiana* is used in diarrhea as it was used for toothache and leucorrhoea in the study of Shah et al. [49]. *A. lebbeck* is species very commonly used in Ayurveda, Siddha and Unani medicinal systems. Local people used this species for wound healing and postnatal care while in literature it was reported as antiallergic, skin tonic and for sexual disorder [49, 90], *C. angustifolia* is used as purgative, hair tonic and teeth tonic whereas in previous study it was used in rheumatism, blood purifier, CNS disorder and joints disorder [3, 47]. *I. linifolia* is used as liver tonic, blood purifier and for stomach complaints while in literature it was reported as amenorrhea [91]. *M. indica* recorded as skin tonic whereas it was reported for respiratory disorder and abdominal pain in the study of Azad Jammu and Kashmir [54]. *M. pudica* is being used as antidote and wound healing but it was reported in literature for menstrual disorder and asthma [86, 92]. Local use of *V. faba* is reported as diuretic while in other regions it was used for kidney pain and eye infection [68]. *A. vera* belonging to family Aleoaceae is used as cough and hair tonic. However previously it was use for sexual and stomach disorder [51, 92]. *M. azadirachta* is effective for wound healing. Already reported use of this species is as tonic for skin and sexual disorders [54, 93]. *F. benghalensis* an important rubber yielding species could also be used for diarrhea while it was already reported in the study of Makerwal and Gulla Khel as blood purifier and for diabetes [49, 94]. *F. religiosa* is locally used for asthma, diarrhea, ulcer, molar pain and cardiac problem as in literature it was reported as laxative and wound healing [55, 95]. Local use of *E. globulus* is used for influenza and stomach disorder but in the previous study it was reported for respiratory disorders [48].

*S. cumini* member of family Myrtaceae is used for the treatment of cancer whereas it was reported for respiratory disorder, ulcer and fever [45, 86]. Efficacy of *A. sativa* for postnatal care is recorded but literature indicates its use as diuretic, laxative, nerve tonic [9, 96]. The medicinal use of *C. barbata* is as skin tonic however it has been used for diabetes [86]. *S. bengalensis* could also be used for postnatal care and animal diseases while it was reported as diuretic too [47]. *T. aestivum*, cereal crop in Pakistan is discovered as anticancer and for GIT but from other regions of Pakistan it was reported for postnatal care and backache [45]. It is discover from present study that *A. arvensis* is good skin and hair tonic and diuretic. However its efficacy is already reported in rheumatism and CNS disorder [76, 86]. *Z. jujube* vernacular name is bari used as skin tonic, hair tonic and also good for joints disorder whereas it was reported for diarrhea, fever and blood purifier [45, 48]. *V. thapsus* is being for sexual disorder and internal bleeding in present study area while it was already reported to be effective as pain killer and used for stomach complaints, respiratory disorder [11, 97]. *L. barbarum* is used as anticancer and for asthma in present study area. but it was reported from Dhinodhar Hill for sexual disorder and skin tonic [89]. *S. americanum* is uncovered for joint disorders, while traditionally it is reported for fever and diarrhea [86]. *W. somnifera* is considered effective for animal diseases in Shahpur Virkan and NavaPind. However according to Kala [47] and Pervaiz [86] this could also be used for treating ulcer and eye infections.

## Conclusions

This study is first quantitative documented information about the traditional uses of wild plants of NavaPind and Shahpur Virkan district Sheikhupura. This survey revealed that this area is rich in wild plants which are medicinally important. Mostly ethnobotanical information in study area is retained by elders, and most of them gained their knowledge from their fore fathers. Unfortunately present generation is not interested to carry this knowledge because of trends of modernization. Hence, there is a gradual loss of traditional knowledge about these plants in new generation. So, it is necessary to document the knowledge for future generation. From this ethnobotanical documented data it can be concluded that the most of plants species having medicinal value belong to family Fabaceae and Poaceae. Both of these families in NavaPind and Shahpur Virkan are represented by fifteen and sixteen species respectively. Both of these families are inhabitant of tropical and subtropical areas. As the most of areas in Punjab have tropical and subtropical conditions. Therefore Fabaceae and Poaceae are not only common in NavaPind and Shahpur Virkan but in the whole Punjab Province. The mode of preparation was decoction followed by extractions from various parts of plants. Quantitative values of ICF, UV, RFC, and FL reflect that study area is blessed with great diversity of medicinal plant and large number of plants are still used by the local people to cure various diseases of human being and animals. The comparative analysis (JI) strengthens the ethnobotanical findings and provides new useful knowledge.it is concluded there were almost 34 plant species reported with different medicinal uses as has been reported previously from different regions of Pakistan and neighboring countries (Table 5). These species were *A. spinosus* (antidote), *A. viridis* (antidote and diuretic), *C. album* (cough), *C. sativum* (joints pain), *C. bonariensis* (asthma and ulcer*), E. alba* (antidote of scorpion), *P. hysterophorus* (blood purifier and for back pain), *S. marianum* (anticancer and wound healing), *B. campestris* (anticancer), *C. reflexa* (wound healing), *C. pepo* (skin tonic), *E. helioscopia* (anticancer and cholera*), R. communis* (wound healing and hair tonic), *A. lebbeck* (wound healing, cough and postnatal care), *C. angustifolia* (purgative, hair tonic and teeth tonic), *D. sissoo* (hair tonic), *M. indica* (skin tonic), *V. faba* (diuretic), *A. vera* (cough and hair tonic), *M. azadirachta* (wound healing), *F. benghalensis* (influenza and diarrhea), *F. religiosa* (asthma, diarrhea, ulcer, molar pain and cardiac problem), *S. cumini* (heart problem), *O. corniculata* (antidote), *A. sativa* (postnatal care), *T. aestivum* (Anticancer and for GIT), *A. arvensis* (skin tonic, hair tonic and diuretic), *R. muricatus* (fever and asthma), *V. thapsus* (male disease and internal bleeding), *L. barbarum* (anticancer and for asthma), *S. americanum* (joints pain), *W. somnifera* (lung disorder, animal disease, male disease and asthma), *U. dioca* (joints pain, cold,flu and liver disease) and *L. camara* (cold and flu). Moreover 12 species (*Carthamus ticntorius*, *Conyza erigeron*, *Schoenoplectus* supinus, *Ocimum sanctum*, *Allium roylei*, *Malva verticillata*, *Malva indica*, *Jasimunum nudiflorum*, *Asthenatherum forkalii*, *Digitalis ciliaris*, *Digitalis nodosa* and *Tetrapagon tenellus*) were reported first time from Pakistan particularly for their medicinal uses. Comparison with ethnobotanical documentation from other Asian and SAARC (South Asian Association for regional Co-operation) countries indicated almost 8 species including *Conyza erigero*, *Allium roylei*, *Malva verticillata*, *Malva indica*, *Jasimunum nudiflorum*, *Digitalis ciliaris*, *Digitalis nodosa* and *Tetrapagon tenellus* were explored first time for their medicinal uses. All these medicinal plants either used in decoction, plaster form or in combination with other medicinal plants or salt for treatment of various diseases (Additional file 1: Table S2).

**Table 5:** Comparison of traditional uses of plant species in the study area with other regions of Pakistan and neighboring country

Sr. No.	Plant species	Uses reported in study area	Uses reported in previous study	References
1	*Andrographis paniculata* (Burm. f.) Wall. ex Nees	Cough, influenza, diarrhea, **blood purifier**	Liver complaints, malaria	[47, 48]
2	*Amaranthus spinosus* L.	Diarrhea, **antidote**, fever, fodder	Eye vision, skin tonic	[48, 68]
3	*Amaranthus viridis* L.	**Constipation**, skin tonic, antidote, diuretic, cooking	Joints pain, burning of feet	[35, 45]
4	*Chenopodium album* L.	**Laxative**, diuretic, cough, cooking	Throat trouble, paralysis, rheumatism, jaundice	[45, 49, 66]
5	*Chenopodium murale* L.	**Laxative**, vegetable, Fodder, dye	Anthelmintic	[72]
6	*Achyranthes aspera* (L.) Hill	**Cough**, laxative, stomach complains, fodder, fuel	Skin tonic, blood purifier, malaria fever	[3, 45, 90]
7	*Coriandrum sativum* L.	**Joint pain**, stomach complaints, cooking	Sexual disease	[100]
8	*Calotropis procera* R. Br.	**Asthma**, skin tonic, fever, fodder	Hair tonic, hepatitis, antidote, tooth tonic	[3, 48, 49, 53, 66]
9	*Artemisia scoparia* Waldst. & Kitt.	**Diuretic**, stomach complains, fodder	Antidote, fever, skin infection	[101]
10	*Conyza bonariensis* L.	**Asthma**, diarrhea, ulcer, fodder	Diuretic, blood purifier	[102, 103]
11	*Eclipta alba* (L.) Hassk.	Liver tonic, hair tonic, **antidote of** **scorpio**, fodder	GIT disorders	[48, 104]
12	*Parthenium hysterophorus* L.	blood purifier, **back pain**, fodder, Decoration, grow in crop	Fever	[45]
13	*Silybum marianum* L.	Anticancer, liver tonic,** wound healing**, fodder	Tuberculosis	[97]
14	*Sonchus arvensis* L.	Anti-kidney stone, **asthma**, cough, fodder	Wound healing	[45]
15	*Xanthium strumarium* L.	Fever, **mother postnatal care**, fodder	Toothache	[72]
16	*Brassica campestris* L.	**Skin tonic**, hair tonic, anti-cancerous	Headache, animal fever	[52, 95]
17	*Brassica nigra* L.	**Diuretic**, respiratory problems, fodder	GIT disease, toothache	[55, 105]
18	*Capsella bursapastoris* L.	Fodder, internal and external bleeding, **diarrhea**	Respiratory disease, sexual disease	[51]
19	*Coronopus didymus* (L.) Sm.	**Asthma**, Blood purifier	Insect repellent, joints disorder	[13, 85]
20	*Sisymbrium irio* L.	Fodder, **asthma**	Fever, skin tonic, cough	[35, 46, 72]
21	*Cannabis sativa* L.	**Stomach complaints**, Relaxant, Severe pains repellent	Male disease	[97]
22	*Convolvulus arvensis* L.	Fodder, **animal fever**, grow in crop	Skin tonic, liver tonic, GIT disorder	[3, 53]
23	*Cuscuta reflexa* Roxb.	**Wound healing**, Hair tonic	Blood purifier, relaxant, fever	[46, 97, 106]
24	*Poranopsis paniculata* (Roxb.) Roberty	Decoration, **stomach pain**, diarrhea, wound healing	Body pain	[50]
25	*Cucurbita pepo* L.	**Skin tonic**, cardiac problem, stomach disorder, Cooking,	Jaundice	[53]
26	*Euphorbia helioscopia* L.	Anticancerous, **cholera**	Skin tonic, GIT disorder, wound healing	[35, 46, 53]
27	*Euphorbia hirta* L.	**Fodder**	Skin tonic, sexual disorder, relaxant, GIT disorder, respiratory disorder	[3, 90, 97, 106]
28	*Euphorbia thymifolia* L.	**Fodder**	Blood pressure	[3]
29	*Ricinus communis* L.	**Wound healing**, fuel, hair tonic	GIT disorder, postnatal care, headache, hepatitis	[45, 48, 53, 98]
30	*Sapium sebiferum* L.	**Antidote**, Fodder, fuel, furniture	GIT disorder	[107]
31	*Acacia.nilotica*	**Fodder**	GIT disorder, sexual disorder wound healing, skin tonic	[3, 46, 98, 106, 108]
32	*Acacia arabica* Willd.	Diarrhea, **Skin tonic**, roof, fuel, fodder, exported	Respiratory disorder	[106]
33	*Acacia farnesiana* L.	**Diarrhea**, perfume, fuel, furniture, exported	Toothache, sexual disorder	[49]
34	*Albizia lebbeck* (L.) Benth.	Fuel, **wound healing**, Cough, burning, postnatal care	Skin tonic	[48, 49]
35	*Cassia angustifolia* L.	**Purgative**, hair tonic, teeth tonic	Blood purifier, sexual disorder, rheumatism	[3, 47]
36	*Cassia fistula* L.	**Laxative**, constipation, fodder, cough, stomach pain	Hepatitis	[48, 90, 106]
37	*Cassia occidentalis* L.	Fuel, roof, **bitter taste**, stomach disorder, laxative	Respiratory disorder	[48]
38	*Dalbergia sissoo* Roxb. ex DC.	Fodder, fuel, furniture, **wound** **healing**, hair tonic	Sexual disease, GIT disease, fever, diuretic	[48, 49]
39	*Indigofera linifolia* (L.f) Retz.	Fodder, obtain dye, Stomach complaints, blood purifier, **for liver**	Sexual disease	[67, 85]
40	*Lathyrus aphaca* L*.*	**Fodder**	Cough	[45]
41	*Melilotus indica* L.	**Laxative**, diarrhea, cooking, fodder, bitter taste, skin tonic	Respiratory disorder, sexual disease	[54, 109]
42	*Mimosa pudica* L.	**Fuel**, fodder, **antidote**, wound healing, diarrhea	Menstrual disorder, respiratory disease	[86, 92]
43	*Tamarindus indica* L.	Malaria, fever, bitter taste, **Laxative**, burning	Jaundice	[48]
44	*Vicia faba* L.	**Diuretic**, fodder, food, soap	Eye infection	[68]
45	*Ocimum basilicum* L.	Scent, flavoring, **cough**, headache, diarrhea	Influenza, cardiac problem, antidote	[53, 104]
46	*Ocimum sanctum* L.	Appetizer, mosquito repellent, fodder, **fever**, cough	Liver tonic	[47, 106, 110]
47	*Aloe vera* (L.) Burm. f.	Skin tonic, cough, hair tonic, **fever**	Liver tonic	[48, 108]
48	*Lawsonia inermis* L.	**Wound healing**, hair tonic	Diuretic, jaundice	[46, 48]
49	*Malvastrum coromandelianum* L.	Fodder, **wound healing**	Fever, GIT disorders	[49]
50	*Azadirachta indica* A. Juss	Fodder, **skin tonic**, fuel	Hair tonic, sexual disorders, eye infection, GIT disorders, liver tonic	[3, 48, 106, 108]
51	*Melia azadirachta* L.	**Wound healing**, shade, cough, fuel, furniture, fodder	Skin tonic, hair tonic	[93]
52	*Ficus sarmentosa* Bush: Ham. ex J.E. Smith	**Fuel**, fodder	Respiratory disease, GIT disorder	[111]
53	*Ficus benghalensis* L.	**Influenza**, shade, diarrhea, fuel, decoction	Asthma, liver tonic	[45, 47, 49]
54	*Ficus religiosa* L.	Asthma, diarrhea Shade, fuel, fodder, ulcer, molar pain, **cardiac** **problem**	Wound healing	[49]
55	*Morus alba* L.	**Cough**, fodder, respiratory disorders	GIT disorder	[11]
56	*Eucalyptus globulus* Labill.	**Influenza**, skin tonic, Fuel, furniture, stomach disorder	Liver tonic	[48]
57	*Syzygium cumini* L.	Fruit, fuel, fodder, cancer, **heart problem**, diarrhea	Nervous disorder, fever	[45, 108]
58	*Oxalis corniculata* L.	**Antidote**, stomach disorder, vegetable, fodder	Liver tonic, diuretic, joints problem	[46, 88]
59	*Fumaria indica* Linn.	**Laxative**, fodder	GIT disorder, liver tonic, diuretic, skin tonic	[45, 53, 93]
60	*Acrachne racemosa* (Roem & schult)	**Fodder**	General debility	[48]
61	*Avena sativa* L.	**Postnatal care**	Nervous disorder, GIT disorders	[53, 97]
62	*Cenchrus setigerus* Vahl.	**Fodder**	Fever, skin tonic, GIT disorder	[66]
63	*Chloris barbata* Sw.	Fodder, **skin disorder**	Respiratory disease	[112]
64	*Chrysopogon aucheri* Bioss	Fodder, **fuel**	GIT disorder	[54]
65	*Cynodon dactylon* (L.) Pers.	Stomach complaint, **constipation**, antidote for snake and dogs, fodder	Diuretic, wound healing, fever	[3, 48, 49, 108]
66	*Eleusine indica* (L.) Gaertn.	**Fodder**	Diuretic, GIT disorder	[96]
67	*Pennisetum divisum* (Fosslk. ex J. F. Gmel)	**Fodder**	GIT disorder	[66]
68	*Phragmites karka* (Retz.) Trin. ex Steud.	**Cardiac problem**, fuel	Respiratory disorder	[16]
69	*Saccharum bengalensis* L.	Roof, fuel, pre/post natal care, **animal disease**	Diuretic	[91]
70	*Triticum aestivum* L.	Fodder, anticancerous, **gastrointestinal disease**	Postnatal care, backache	[45]
71	*Rumex chalepensis* Mill.	**Fodder**	Insect repellent	[113]
72	*Anagallis arvensis* L.	Fodder, **skin tonic, Diuretic**, hair tonic	Nervous disorder	[90, 104]
73	*Ranunculus muricatus* L.	Fodder, fever, **asthma, Fodder**	Wound healing, skin tonic	[45, 46]
74	*Ranunculus repens* L.	**Fodder**	Nervous disorder, blood purification	[84]
75	*Zizyphus jujuba* (L.) Lam.	Fruit, furniture, skin tonic, joints pain, hair tonic, **backache**, burning	Liver tonic, GIT disorder	[45, 48]
76	*Murraya koenigii* L.	Stomach diseases, wound healing, vomiting, scent, **decoration**	Blood purification	[86]
77	*Salix lanata* L.	Fever, **reduce internal pain**, fodder, bitter taste	Respiratory disorder	[3]
78	*Verbascum thapsus* L.	**Male disease**, internal bleeding	Wound healing, nervous disorder, respiratory disorder	[11, 49]
79	*Lycium barbarum* L.	**Anticancerous**, asthma, scent, fodder	Diuretic, skin tonic	[89, 114]
80	*Solanum americanum* Mill.	**Joints pain**, cooking, **Perfume**, vegetable, fodder	GIT disorders. fever	[48]
81	*Withania somnifera* L.	Lung disorder, **animal disease**, asthma, male disease	Fever, joint pain, liver tonic, sexual disorder, eye infection	[45–47, 53, 66, 108]
82	*Urtica dioica* L.	**Joints pain**, cold and flu, liver disease	Skin tonic, GIT disorder, fever	[91, 115]
83	*Lantana camara* L.	Fodder, snake bite, **cold and flu**	Malaria fever	[49]
84	*Phyla nodiflora* L.	**Fodder**	GIT disorder, diuretic, blood purifier, wound healing, hair tonic	[45, 46, 49, 104]

Fl value is calculated for Bold diseases

The traditional medicine used in the region lacks physiotherapeutic evidence. It is necessary to perform phytochemical and pharmacological studies to explore the potential of such plants herbal drug discovery. This study also provides basis for the conservation of the local flora. It will also provide various socio-economic dimensions associated with the common people. Further strategies should be taken for conservation of these medicinal plants on priority in NavaPind and ShahpurVirkan, Sheikhupura, Pakistan.

## Additional file

Additional file 1:
**List S1.** Names of plant species with accession numbers. **Table S1.** Preparation and administration of various herbal remedies frequently used in NavaPind and Shahpur Virkan, district Sheikhupura, Province Punjab, Pakistan. **Annex S1.** Questionnaire. (DOCX 46 kb)
