# Supplementary material for: An ethnopharmacological evaluation of Navapind and Shahpur Virkanin district Sheikupura, Pakistan for their herbal medicines
Source: J Ethnobiol Ethnomed. 2017 May 8;13:27. doi: 10.1186/s13002-017-0151-1 (PMC5422909; doi:10.1186/s13002-017-0151-1)
Supplement: Additional file 1: — List S1. Names of plant species with accession numbers. Table S1. Preparation and administration of various herbal remedies frequently used in NavaPind and Shahpur Virkan, district Sheikhupura, Province Punjab, Pakistan. Annex S1. Questionnaire. (DOCX 46 kb) [file 13002_2017_151_MOESM1_ESM.docx]

**AN ETHNOPHARMACOLOGICALEVALUATION OF NAVAPIND AND SHAHPUR VIRKANIN DISTRICT SHEIKUPURA, PAKISTAN FOR THEIR HERBAL MEDICINES.**

Maria Zahoor, *Zubaida Yousaf, Tahreem Aqsa, Manahil Haroon, Nadia Saleh, Arusa Aftab, Sadia Javed, Mouzma Qadeer and Habiba Ramazan

Department of Botany, Lahore College for women university, Lahore Pakistan

CorespondingautherEmail: *[z4zubaida@yahoo.com](mailto:z4zubaida@yahoo.com) and [mussab.wajahat@gmail.com](mailto:mussab.wajahat@gmail.com)

Emails of coauthors: maria.zahoor12345@gmail.com

[Taqsa1236@gmail.com](mailto:Taqsa1236@gmail.com)

[haroon@yahoo.com](mailto:haroon@yahoo.com)

[nadiasaleh14@yahoo.com](mailto:nadiasaleh14@yahoo.com)

[arusa_ravi@yahoo.com](mailto:arusa_ravi@yahoo.com)

[jsadia93@gmail.com](mailto:jsadia93@gmail.com)

[mouzma@hotmail.com](mailto:mouzma@hotmail.com)

[habibalcwu0@gmail.com.](mailto:habibalcwu0@gmail.com.)

**Abstract:**

***Background:*** The chief aim of this study was to enlist the ethnobotanical uses of wild plants in district Sheikhupura, province Punjab, Pakistan. Due to its extreme geographical and climatic conditions, Pakistan has a great floral diversity. Plants have been used by the indigenous people for the treatment of different ailments since long time. They still depend on the plants for their domestic purposes. Moreover the plants are used as first aid to treat different ailments such as cold, cough, influenza, asthma, cancer, antidote, gastric and hepatic disorders. The traditional uses of medicinal plants lead to the discovery of natural drugs. This is basically the first quantitative ethnobotanical documentation of medicinal plants in NavaPind and ShahpurVirkan district Sheikhupura, province Punjab, Pakistan.

***Materials and Method:*** This ethnobotanical information was collected from the 400 informants including male and female. The informative data was based on semi-structured interviews, group discussions, and ﬁeld visits. Then the data was analyzed by applying different quantitative indices such as informant consent factor (ICF), Use value (UV), Relative Frequency of Citation (RFC), the Fidelity level (FL) and Jaccard Index (JI).

***Result:*** Almost 96 plants belonging to 34 families were reported. Most-frequently cited families were the Poaceae (16 species) and Fabaceae (ten species). Herbs were the most dominant life form (30.20%). Leaves were the most-used plant parts (31.14%), followed by whole plant (24.59%). Extract was the common mode of preparation reported (81.25%). Mostly herbal medicines were acquired from fresh plant material. Among all 54.16% plants were toxic, 31.25% nontoxic, whereas the remaining 14.58% may be toxic or nontoxic because of their dual attitude. Almost 34 species are reported with their different medicinal uses as has been reported previously.

***Conclusion:*** This ethnobotanical documentation revealed that the plants are still used by natives of rural area in their day-to-day lives. This study provides the basis for the conservation of local flora. Plants with high ICF, UV and FL were further used for phytochemical and pharmacological studies. This documentation is baseline information which can be used to develop new plant-based commercial drugs.

Keywords: Ethnobotany, Fabaceae, Medicinal plant, Poaceae, Traditional knowledge

**List S1: Names of plant species with accession numbers**

| **Sr. no.** | **PLANTS NAME/FAMILY** | **ACCESSION NUMBER** |
| --- | --- | --- |
|  | *Andrographis paniculata* (Burm. f.) Wall. ex Nees  Acanthaceae | LCWU-15-01. |
|  | *Amaranthus spinosus* L.  Amaranthaceae | LCWU-15-02 |
|  | *Amaranthus viridis* L.  Amaranthaceae | LCWU-15-03 |
|  | *Chenopodium album* L.  Chenopodiaceae | LCWU-15-04 |
|  | *Chenopodium murale* L.  Chenopodiaceae | LCWU-15-05 |
|  | *Achyranthes aspera* (L.) Hill  Amaranthacea | LCWU-15-06 |
|  | *Coriandrum sativum* L.  Apiaceae | LCWU-15-07 |
|  | *Calotropis procera* R. Br.  Asclepiadaceae | LCWU-15-08 |
|  | *Artemisia scoparia* Waldst. & Kitt.  Asteraceae | LCWU-15-0902 |
|  | *Carthamus ticntorius* L.  Asteraceae | LCWU-15-10 |
|  | *Conyza bonariensis* L.  Asteraceae | LCWU-15-1102 |
|  | *Conyza erigeron* L.  Asteraceae | LCWU-15-1202 |
|  | *Eclipta alba* (L.) Hassk.  Asteraceae | LCWU-15-13 |
|  | *Parthenium hysterophorus* L.  Asteraceae | LCWU-15-14 |
|  | *Silybum marianum* L.  Asteraceae | LCWU-15-1501 |
|  | *Sonchus arvensis* L.  Asteraceae | LCWU-15-1601 |
|  | *Xanthium strumarium* L.  Asteraceae | LCWU-15-17 |
|  | [Brassica](http://plants.usda.gov/java/ClassificationServlet?source=display&classid=CYDA) *campestris* L.  Brassicaceae | LCWU-15-18 |
|  | *Brassica nigra* L.  Brassicaceae | LCWU-15-19 |
|  | *Capsella bursa­-pastoris* L.  Brassicaceae | LCWU-15-20 |
|  | *Coronopus didymus* (L.) Sm.  Brassicaceae | LCWU-15-2003 |
|  | *Sisymbrium irio* L.  Brassicaceae | LCWU-15-96 |
|  | *Cannabis sativa* L.  Cannabiaceae | LCWU-15-22 |
|  | *Convolvulus arvensis* L.  Convolvulaceae | LCWU-15-23 |
|  | *Cuscuta reflexa* Roxb.  Convolvulaceae | LCWU-15-24 |
|  | *Poranopsis paniculata* (Roxb.) Roberty  Convolvulaceae | LCWU-15-25 |
|  | *Cucurbita pepo* L.  Cucurbitaceae | LCWU-15-2601 |
|  | *Schoenoplectus supinus* (L.) Pall.  Cyperaceae | LCWU-15-27 |
|  | *Euphorbia helioscopia* L.  Euphorbiaceae | LCWU-15-28 |
|  | *Euphorbia hirta* L.  Euphorbiaceae | LCWU-15-29 |
|  | *Euphorbia thymifolia* L.  Euphorbiaceae | LCWU-15-30 |
|  | *Ricinus communis* L.  Euphorbiaceae | LCWU-15-3101 |
|  | *Sapium sebiferum* L.  Fabaceae | LCWU-15-32 |
|  | *Acacia* *nilotica* (Linn.) Delile.  Fabaceae | LCWU-15-93 |
|  | *Acacia Arabica* Willd.  Fabaceae | LCWU-15-3301 |
|  | *Acacia farnesiana* L.  Fabaceae | LCWU-15-34 |
|  | *Albizia lebbeck* (L.) Benth.  Fabaceae | LCWU-15-3501 |
|  | *Cassia angustifolia* L.  Fabaceae | LCWU-15-3602 |
|  | *Cassia fistula* L.  Fabaceae | LCWU-15-37 |
|  | *Cassia occidentalis* L.  Fabaceae | LCWU-15-38 |
|  | *Dalbergia sissoo* Roxb. ex DC.  Fabaceae | LCWU-15-3901 |
|  | *Indigofera linifolia* (L. f.) Retz.  Fabaceae | LCWU-15-40 |
|  | *Lathyrus aphaca* L*.*  Fabaceae | LCWU-15-41 |
|  | *Melilotus indica* L.  Fabaceae | LCWU-15-4202 |
|  | *Mimosa pudica* L.  Fabaceae | LCWU-15-94 |
|  | *Tamarindus indica* L.  Fabaceae | LCWU-15-4301 |
|  | *Vicia faba* L.  Fabaceae | LCWU-15-4402 |
|  | *Ocimum basilicum* L.  Lamiaceae | LCWU-15-45 |
|  | *Ocimum sanctum* L.  Lamiaceae | LCWU-15-4601 |
|  | *Allium roylei* Strearn.  Liliaceae | LCWU-15-47 |
|  | *Aloe vera* (L.) Burm. f.  Asphodelaceae | LCWU-15-48 |
|  | *Lawsonia inermis* L.  Lythraceae | LCWU-15-49 |
|  | *Malva verticillata* L.  Malvaceae | LCWU-15-50 |
|  | *Malva indica*  Malvaceae | LCWU-15-51 |
|  | *Malvastrum coromandelianum* L.  Malvaceae | LCWU-15-52 |
|  | *Azadirachta indica* A. Juss  Meliaceae | LCWU-15-53 |
|  | *Melia azadirachta* L.  Meliaceae | LCWU-15-5402 |
|  | *Ficus* *sarmentosa* Bush: Ham. ex J.E. Smith  Moraceae | LCWU-15-95 |
|  | *Ficus benghalensis* L.  Moraceae | LCWU-15-5501 |
|  | *Ficus religiosa* L.  Moraceae | LCWU-15-56 |
|  | *Morus alba* L.  Moraceae | LCWU-15-5701 |
|  | *Eucalyptus globules* Labill.  Myrtaceae | LCWU-15-58 |
|  | *Syzygium cumini* L.  Myrtaceae | LCWU-15-59 |
|  | *Jasminum nudiflorum* Lindl.  Oleaceae | LCWU-15-6002 |
|  | *Oxalis corniculata* L.  Oxalidaceae | LCWU-15-6101 |
|  | *Fumaria indica* L.  Papaveraceae | LCWU-15-6202 |
|  | *Acrachne racemosa* (Roem & schult)  Poaceae | LCWU-15-63 |
|  | *Asthenatherum forkalii* (Vahl) Nexski.  Poaceae | LCWU-15-64 |
|  | *Avena sativa* L.  Poaceae | LCWU-15-65 |
|  | *Cenchrus setigerus* Vahl.  Poaceae | LCWU-15-66 |
|  | *Chloris barbata* Sw.  Poaceae | LCWU-15-67 |
|  | *Chloris virgata* Sw.  Poaceae | LCWU-15-68 |
|  | *Chrysopogon aucheri* Bioss  Poaceae | LCWU-15-69 |
|  | [Cynodon dactylon (L.) Pers.](http://plants.usda.gov/java/ClassificationServlet?source=display&classid=CYDA)  Poaceae | LCWU-15-70 |
|  | *Digitaria ciliaris* (Retz.) Koel.  Poaceae | LCWU-15-71 |
|  | *Digitaria nodosa* Parl.  Poaceae | LCWU-15-72 |
|  | *Eleusine indica* (L.) Gaertn.  Poaceae | LCWU-15-73 |
|  | *Pennisetum divisum* (Fosslk. ex J. F. Gmel)  Poaceae | LCWU-15-74 |
|  | *Phragmites karka* (Retz.) Trin. ex Steud.  Poaceae | LCWU-15-75 |
|  | *Saccharum bengalensis* L.  Poaceae | LCWU-15-76 |
|  | *Tetrapogon tenellus* (Roxb.) Chiov.  Poaceae | LCWU-15-77 |
|  | *Triticum aestivum* L.  Poaceae | LCWU-15-78 |
|  | *Rumex chalepensis* Mill.  Polygonaceae | LCWU-15-79 |
|  | *Anagallis arvensis* L.  Primulaceae | LCWU-15-8001 |
|  | *Ranunculus muricatus* L.  Ranunculaceae | LCWU-15-8101 |
|  | *Ranunculus repens* L.  Ranunculaceae | LCWU-15-82 |
|  | *Zizyphus jujube* (L.) Lam.  Rhamnaceae | LCWU-15-8301 |
|  | *Murraya koenigii* L.  Rutaceae | LCWU-15-84 |
|  | *Salix lanata* L.  Salicaceae | LCWU-15-85 |
|  | *Verbascum thapsus* L.  Scrophulariaceae | LCWU-15-86 |
|  | *Lycium barbarum* L.  Solanaceae | LCWU-15-87 |
|  | *Solanum americanum* Mill.  Solanaceae | LCWU-15-88 |
|  | *Withania somnifera* L.  Solanaceae | LCWU-15-89 |
|  | *Urtica dioca* L.  Urticaceae | LCWU-15-90 |
|  | *Lantana camara* L.  Verbenaceae | LCWU-15-9101 |
|  | *Phyla nodiflora* L.  Verbenaceae | LCWU-15-92 |

**Table S2: Preparation and administration of various herbal remedies frequently used in NavaPind and Shahpur Virkan, district Sheikhupura, Province Punjab, Pakistan.**

| **SR. NO.** | **PLANT SPECIES** | **MODE OF ADMINISTRATION** |
| --- | --- | --- |
|  | *Andrographis paniculata* (Burm. f.) Wall. ex Nees | Joshanda is used for flu and cough by soaking in the water overnight |
|  | *Amaranthus spinosus* L. | Peel the leaves to extract the juice and used as antidote |
|  | *Amaranthus viridis* L. | Peel the leaves to extract the juice add some salt. This juice is effective in constipation Externally the paste of leaves used as antidote.. |
|  | *Chenopodium album* L. | Leaves extract with Neem leaves is used for urinary problem. |
|  | *Chenopodium murale* L. | Decoction is drunk for stomach complaints. |
|  | *Achyranthes aspera* (L.) Hill | Decoction is used for laxative and also in cough |
|  | *Coriandrum sativum* L. | Boil seeds in water and drink the solution for digestion |
|  | *Calotropis procera* R. Br. | Decoction is used for asthma and latex of this specie is used as skin tonic. |
|  | *Artemisia scoparia* Waldst. & Kitt. | Oil of plant species is used taken as diuretic and some digestion complaints. |
|  | *Carthamus tinctorius* L. | Boil the leaves with water and make extract. Put few drops of this extract on wound. |
|  | *Conyza bonariensis* L. | Use of extract for asthma and ulcer. |
|  | *Conyza erigeron* L. | Decoction is drunk for respiratory problems. It also aids in digestion. |
|  | *Eclipta alba* (L.) Hassk. | Paste of leaves is used against the bite of scorpio. |
|  | *Parthenium hysterophorus* L. | Drink the extract of leaves for pain. It also helps to purify the blood. |
|  | *Silybum marianum* L. | Drink decoction of this plant, will inhibit the formation of tumor and also detoxify the harmful substances in liver. |
|  | *Sonchus arvensis* L. | Use the extract of this plant prevents the formation of stone in kidney and sort out some respiratory problems. |
|  | *Xanthium strumarium* L. | Taking extract of leaves, will help in delivery. |
|  | [Brassica](http://plants.usda.gov/java/ClassificationServlet?source=display&classid=CYDA) *campestris* L. | Extract/oil is used for healthy hair, by mixing mustard oil, besan, curd and few drops of lemon juice for dark spots on skin. |
|  | *Brassica nigra* L. | Decoction of this plant helps in urination. |
|  | *Capsella bursa­pastoris* L. | Decoction is used for diarrhea and other stomach complaints. Also control the internal bleeding. |
|  | *Coronopus didymus* (L.) Sm. | Few drops of extract, will act as blood purifier. |
|  | *Sisymbrium irio* L. | Extract of this plant is used for respiratory diseases i.e. asthma, cough. |
|  | *Cannabis sativa* L. | Crush the leaves and make a paste. Take this paste with milk to relieve pain i.e. headache |
|  | *Convolvulus arvensis* L. | Boil the leaves with water and give it to animal for animal fever. |
|  | *Cuscuta reflexa* Roxb. | Boil the leaves to make extract. Put few drops of extract on wound for healing. |
|  | *Poranopsis paniculata* (Roxb.) Roberty | Use extract to prevent stomach pain and make digestion easy. It is also used for wound healing. |
|  | *Cucurbita pepo* L. | Rub the extract on skin to prevent different skin itching. It also aid in heart problems. |
|  | *Euphorbia helioscopia* L. | Decoction of seeds inhibits the formation of cancer cells and also used in cholera. |
|  | *Ricinus communis* L. | Oil/extract is used to soften hair, rub oil on nails for breaking nails. |
|  | *Sapium sebiferum* L. | Decoction of leaves is used as an antidote. |
|  | *Acacia arabica* Willd. | Decoction of bark is used for skin itching and skin rashes. It also helps in diarrhea. |
|  | *Acacia farnesiana* L. | Drink the extract in diarrhea to fix it. |
|  | *Albizia lebbeck* (L.) Benth. | Put few drops of extract on wound to heal it. |
|  | *Cassia angustifolia* L. | Soak the leaves overnight in water and wash the hair with that water. It strengthens the hair and prevents hair falling. |
|  | *Cassia fistula* L. | 1 teaspoon amaltas fruit pulp + 1 teaspoon of tamarind in one cup of water left overnight mashed and strained is used for stomach problem. |
|  | *Cassia occidentalis* L. | Drink the extract to make digestion easy and for other stomach complaints. |
|  | *Dalbergia sissoo* Roxb. ex DC. | Sap of extract used for wound healing and dark spots. Juice with honey for eye pain. |
|  | *Indigofera linifolia* (L.f) Retz. | Drink the extract to relieve stomach pain and it also acts as blood purifier. |
|  | *Melilotus indica* L. | Use the extract helps in diarrhea and sometime also used as laxative |
|  | *Mimosa pudica* L. | Decoction used for stomach complaints. Leaves liquor used for wound healing |
|  | *Tamarindus indica* L. | Leaves extract mix with sugar used for digestive diseases |
|  | *Vicia faba* L. | Extract of leaves helps in urination. |
|  | *Ocimum basilicum* L. | Due to its fragrance, extract is used as scent. It also helps to eradicate headache. |
|  | *Ocimum sanctum* L. | Rub the oil of this plant on body as it is used as mosquito repellent. |
|  | *Allium roylei* Strearn. | Drink extract in cholera. Oil of this plant is used as flavor in food. |
|  | *Aloe vera* (L.) Burm. f. | Gum of leaves is applied on skin to smoothen the skin and freshen it up. While oil is applied in hair roots to strengthen hairs |
|  | *Lawsonia inermis* L. | Powder mix with water to make paste for hair dye. |
|  | *Malva indica* | Decoction is used for cough. |
|  | *Malvastrum coromandelianum* L. | Put few drops of extract on wound to heal it. |
|  | *Azadirachta indica* A. Juss | Leaves powder with water is used for diabetes. Leaves extract is used for skin problems |
|  | *Melia azadirachta* L. | Boil in water and wash hairs with this water for healthy hair. It is also used for wound healing |
|  | *Ficus sarmentosa* Bush: Ham. ex J.E. Smith | Decoction |
|  | *Ficus benghalensis* L. | Decoction of plant is used for diarrhea. |
|  | *Ficus religiosa* L. | Decoction is used for heart problem and for diarrhea. |
|  | *Morus alba* L. | Extract of plant is taken for respiratory diseases. |
|  | *Eucalyptus globulus* Labill. | Infusion of leaves used for skin disease. |
|  | *Syzygium cumini* L. | Mix black pulm pulp, barley flour, Indian gooseberry juice and rose water as a face pack for oily skin |
|  | *Jasminum nudiflorum* Lindl. | Few drops of extract is added in food to get aroma. |
|  | *Oxalis corniculata* L. | Boil the leaves in water and make extract. Put a drop of juice (extract) on bite of snake/scorpio. |
|  | *Fumaria indica* Linn. | Extract of leaves is used as laxative and for stomach pain. |
|  | *Avena sativa* L. | Soak the leaves in water overnight and drink the water for postnatal care and ease in delivery. |
|  | *Chloris barbata* Sw. | Peel the leaves and crush them then apply on skin for different skin itching and other skin disorders. |
|  | *Chloris virgata* Sw. | Boil the leaves in water and put few drops in wound. |
|  | [Cynodon dactylon (L.) Pers.](http://plants.usda.gov/java/ClassificationServlet?source=display&classid=CYDA) | Boil the leaves with water to make extract. Drink this extract juice to prevent stomach complaints. |
|  | *Phragmites karka* (Retz.) Trin. ex Steud. | Soak leaves in water overnight and drink this extract to avoid heart problem. |
|  | *Saccharum bengalensis* L. | Paste of plant is used on stitches of delivery and also applies on animal for different diseases. |
|  | *Triticum aestivum* L. | Chapatti used to increase weight and for constipation |
|  | *Anagallis arvensis* L. | Extract is drunk for skin disorder and to make urination ease. |
|  | *Ranunculus muricatus* L. | Boil leaves in water and |
|  | *Zizyphus jujuba* (L.) Lam. | Extract combine with ginger or mint for achy throat muscles. Its oil is used for joints pain |
|  | *Murraya koenigii* L. | Put few drops of extract on wound to heal it. Also drink for stomach diseases. |
|  | *Salix lanata* L. | Boil leaves in water in fever to recover. |
|  | *Verbascum thapsus* L. | Infusion is taken to develop maleness in men. |
|  | *Lycium barbarum* L. | Extract is taken against cancer. |
|  | *Solanum americanum* Mill. | Stem extract is rub on joints to relieve pain. |
|  | *Withania somnifera* L. | Extract is used to improve maleness in men and some lung disorders. |
|  | *Urtica dioica* L. | Soak leaves in water overnight and drink for influenza and cough. |
|  | *Lantana camara* L. | Put few drops on bite of snake and scorpio that acts as antidote. |

**Annexure: S1**

**QESTIONAIRE**

**Lahore College for Women University, Lahore**

**Department of Botany**

- Participant # or Name:--------------------------------------------------
- Participant Age:___________________________________
- Participant Religion________________________________
- Participant Highest level of education:------------------------------
- Participants home language:--------------------------------------------------------------
- Participants Ethnic identity:--------------------------------------------------------------
- **Data about medicinal plant and its use**
- Plant (Local name):---------------------------------------------------------------------------
- Habit (Tree/herb/shrub/climber):--------------------------------------------------------
- Plant part used:-------------------------------------------------------------------------------
- Cultivated/wild:-------------------------------------------------------------------------------
- If cultivated, cultivated for:----------------------------------------------------------------
- If wild, availability in natural resources

a: easy, b: difficulty, c: very difficult :--------------------------------------------------

- Conservation needs :------------------------------------------------------------------------
- Method of collection and storage :----------------------------------------------------------------------------------------------------------------------------------------------------------
- Name of diseases treated :-----------------------------------------------------------------
- Mode of Administration :-------------------------------------------------------------------
- Other uses
- **Questions**
- What plant foods have you eaten in the last three days?

--------------------------------------------------------------------------------------------------------------------------------------------------------------------------------------------------------

- Which of the plant foods eaten in the last three days were grown locally?

----------------------------------------------------------------------------------------

- What are the most important plants foods that are eaten during festivals?

--------------------------------------------------------------------------------------------------------------------------------------------------------------------------------------------------------

- When members of your family are sick, where do you seek advice and treatment? Do you seek help from a doctor/health clinic, a traditional healer, or do you use home remedies?

--------------------------------------------------------------------------------------------------------------------------------------------------------------------------------------------------------

- If you use home remedies or a traditional healer, what kind of sickness is regularly treated? What kinds of plants are used in to treat each sickness?

--------------------------------------------------------------------------------------------------------------------------------------------------------------------------------------------------------

- What other plants are used on a daily basis for health care?

--------------------------------------------------------------------------------------------------------------------------------------------------------------------------------------------------------

- When cooking food or heating your home what do you use for fuel?

----------------------------------------------------------------------------------------------------

- If fires are made with wood or other plants, what are the best fires making materials in your area?

--------------------------------------------------------------------------------------------------------------------------------------------------------------------------------------------------------

- Does your family grow any plants for food, medicine or fuel? if so, what kinds of plants are grown for food, medicine and fuel ?

--------------------------------------------------------------------------------------------------------------------------------------------------------------------------------------------------------

- For plants reported as being grown, which are grown inside the home, in a home garden, in places away from your home?

----------------------------------------------------------------------------------------------------

- When you were growing up, did your family have a garden? if yes, what kinds of things did you grow ?

----------------------------------------------------------------------------------------------------

- Did you have a favorite (plant) food? what other kinds of food did you eat?

----------------------------------------------------------------------------------------------------

- Did you ever gather fruits from trees in your yard or in the forest? if yes, what kinds ? Where were they growing?

--------------------------------------------------------------------------------------------------------------------------------------------------------------------------------------------------------

- Did you know any superstitions or myths about certain plants?

--------------------------------------------------------------------------------------------------------------------------------------------------------------------------------------------------------

- Are there any plants that are special for you here in Soon Sakesar valley?

--------------------------------------------------------------------------------------------------------------------------------------------------------------------------------------------------------

- other information, if any

----------------------------------------------------------------------------------------------------------------------------------------------------------------------------------------------------------------------------------------------------------------------------------------------------------------------------------------------------------------
